# Native alleles at *lhcb6* shape photosynthetic efficiency and early growth in maize

**DOI:** 10.1038/s41598-026-42348-8

**Published:** 2026-03-06

**Authors:** Sebastian Urzinger, Lukas Würstl, Viktoriya Avramova, Claude Urbany, Daniela Scheuermann, Thomas Presterl, Stefan Reuscher, Manfred Mayer, Sarah Brajkovic, Bernhard Küster, Milena Ouzunova, Bernardo Ordas, Peter Westhoff, Chris-Carolin Schön

**Affiliations:** 1 Plant Breeding, TUM School of Life Sciences, Technical University of Munich, 85354 Freising, Germany; 2 Maize Breeding, KWS SAAT SE & Co. KGaA, 37574 Einbeck, Germany; 3 Present Address: Bayer Crop Science, 46325 Borken, Germany; 4 Proteomics and Bioanalytics, TUM School of Life Sciences, Technical University of Munich, 85354 Freising, Germany; 5 Misión Biológica de Galicia, Spanish National Research Council (CSIC), Pontevedra, 36080 Spain; 6 Institute of Molecular and Developmental Biology of Plants, Heinrich-Heine-University Düsseldorf, 40225 Düsseldorf, Germany

**Keywords:** Biotechnology, Genetics, Plant sciences

## Abstract

Maize is a globally important staple crop, providing food, animal feed, and industrial raw materials. Its extensive native diversity can be utilized to broaden the genetic basis for quantitative trait improvement and advance our understanding of the genotype-phenotype relationships underlying complex trait variation. In a forward genetic approach, we dissect a locus on chromosome 10 with major effects on Photosystem II (PSII) maximum quantum efficiency (F_v_/F_m_), non-photochemical quenching (NPQ) and biomass accumulation during early growth stages. Integrating molecular and physiological information we show that allelic variation at the gene encoding LIGHT HARVESTING CHLOROPHYLL A/B BINDING PROTEIN6 (LHCB6 also known as CP24), a component of the PSII light-harvesting complex (LHCII) antenna, underlies this locus. We demonstrate that the allelic variation results from a hAT transposon insertion at *lhcb6* and is associated with decreased accumulation of the LHCII antenna components LHCB6 and LHCB3. Based on proteomic analyses we propose candidate genes that partially compensate for the unfavorable early growth effects caused by impaired LHCII antenna assembly. Our work provides novel insights into the function of *lhcb6* in the C_4_ crop maize and demonstrates the value of natural variation for the understanding and genetic improvement of complex photosynthetic processes.

**Supplementary Information:**

The online version contains supplementary material available at 10.1038/s41598-026-42348-8.

†These authors contributed equally (SU and LW).

## Introduction

Crop yields have increased steadily in recent decades, yet future genetic improvement will require broadening the genetic base of elite crop germplasm^1,2^. In maize (*Zea mays* L.), multiple genetic bottlenecks—starting with its geographic expansion from its center of origin, followed by the establishment of heterotic groups using a small number of landraces, and subsequent cycles of intense selection—have significantly narrowed the genetic diversity of elite germplasm^3–5^. Thus, beneficial alleles that influence (a)biotic stress tolerance, resource efficiency and photosynthetic traits, likely have been lost over time and undesirable alleles might have been fixed through genetic drift or linkage drag^6–8^. Breakthroughs in genetic engineering have deepened our understanding of plant physiology and raised hopes for the development of improved crops. However, when it comes to quantitative traits, translating molecular advances to field performance often falls short of expectations^9,10^. Alternatively, landraces offer a wealth of untapped allelic variation that can be leveraged to improve agronomically and ecologically significant traits^1,11–13^. Despite their conceptual value, the incorporation of landraces into breeding programs is hindered by their yield gap compared to elite lines^11,14,15^ and the risk of linkage drag due to the co-inheritance of undesirable and beneficial alleles^16,17^. One strategy to overcome these challenges is to extract clearly defined alleles associated with quantitative traits of interest from preselected landraces^12^. In a previous study, we reported a successful example for such an approach by demonstrating that favorable allelic variation at the *ndhm1* locus, modulating cyclic electron transport, has pleiotropic positive effects on photosystem II (PSII) efficiency, cold tolerance and plant growth^18^.

Here, we focus on the target trait maximum quantum efficiency of PSIIF_v_/F_m_). F_v_/F_m_ provides an estimate of how efficient light energy is used for photochemistry. In unstressed leaves F_v_/F_m_ is stable, and a reduction in F_v_/F_m_ is generally indicative of stress^19^. In maize, genetic variation in F_v_/F_m_ has been shown to be correlated with multiple traits such as early plant development, chilling tolerance, PSII operating efficiency (ΦPSII), non-photochemical quenching (NPQ) and chlorophyll content^11,20,21^. Thus, F_v_/F_m_ can serve as a proxy for assessing genetic variation related to stress tolerance^22^, but it is also related to biomass production by modulation of light interception- and conversion-efficiency^9,19,23,24^. Previous studies using biparental or multi-parent advanced generation inter-cross (MAGIC) populations have identified only a few quantitative trait loci (QTL) for F_v_/F_m_, each with relatively large effects^20,25–28^. Candidate genes have been proposed for these QTL, but the physiological mechanisms underlying the variation of F_v_/F_m_ in maize have remained largely unclear.

In this study we performed a comprehensive analysis of QTL for F_v_/F_m_ in a population of 222 doubled-haploid (DH) lines derived from the maize landrace “Kemater Landmais Gelb” (Kemater). First, we conducted a genome-wide association study (GWAS), to explore the genetic architecture of F_v_/F_m_ variation in the landrace. We then related the genetic variation in F_v_/F_m_ to physiological parameters significantly influencing agronomically important traits. Our analysis led to the discovery of a genomic region on maize chromosome 10 harboring allelic variation at *light harvesting chlorophyll a/b binding protein6* (*lhcb6* also known as *CP24*), a gene encoding a component of the PSII light-harvesting complex, as a key determinant of F_v_/F_m_ variation in Kemater. We subsequently characterized the impact of this allelic variation on transcript levels and the leaf proteome, as well as on the physiological trait NPQ and plant growth. We also investigated the prevalence of specific alleles at this gene in diverse maize inbred lines.

## Results

### Discovery of QTL affecting F_v_/F_m_ in a maize landrace

**Fig. 1:**
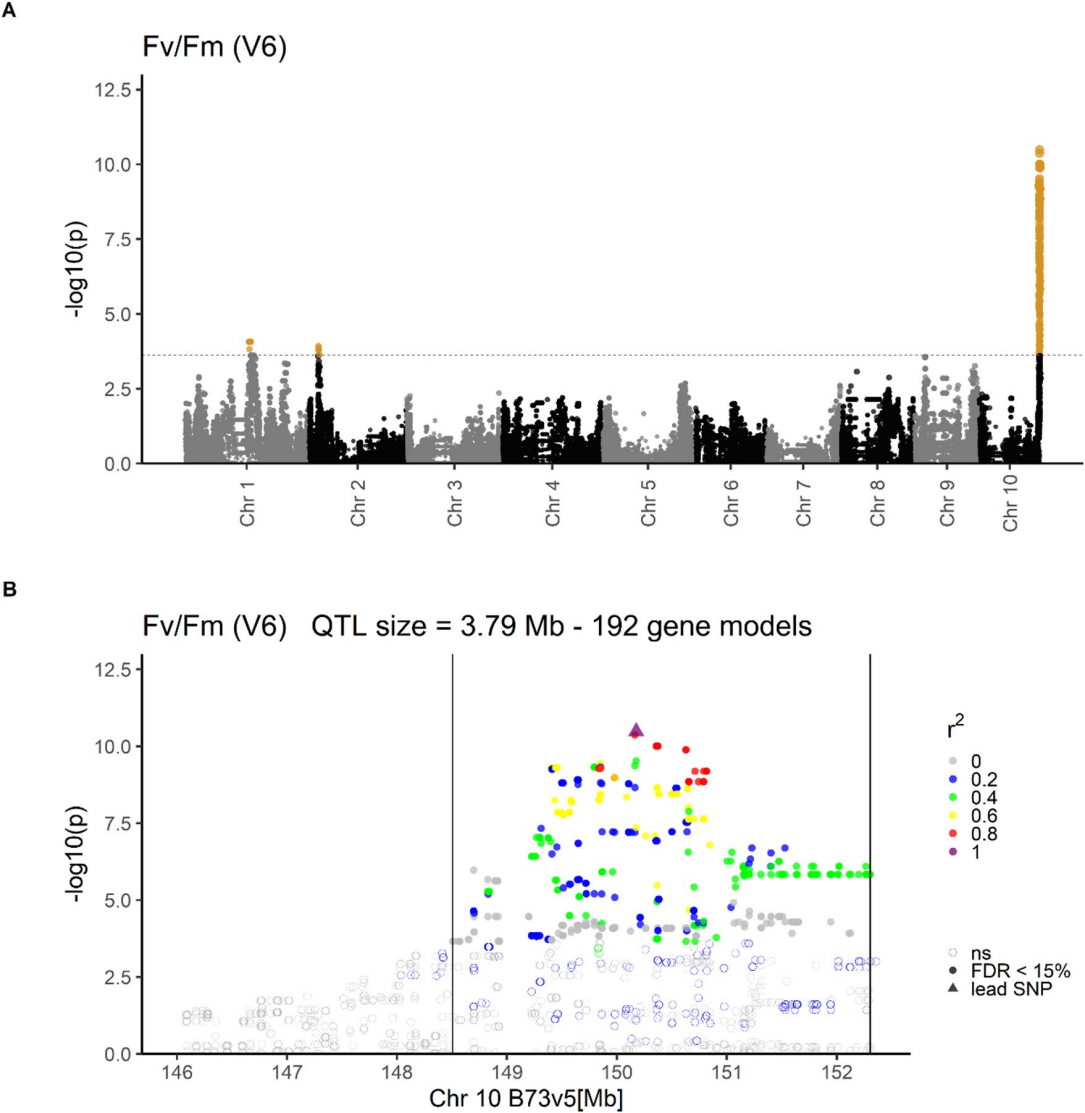
Marker-trait associations from a genome-wide association study (GWAS) for maximum potential quantum yield of photosystem II (F_v_/F_m_) in growth stage V6. **(A)** Marker-trait associations across chromosomes. Phenotypic data were assessed on 211 DH lines derived from the landrace Kemater during early development in growth stage V6 in field experiments in two locations. The x-axis shows the ten chromosomes of maize and the y-axis the -log10 of the p-value of associations of SNPs with F_v_/F_m_. The significance threshold (false discovery rate FDR < 15%) is indicated by a dashed horizontal line. Marker-trait associations are color-coded: orange (significant); grey and black (not significant, ns). **(B)** Marker-trait associations for the QTL on chromosome 10. The x-axis indicates the physical position of SNPs on chromosome 10 in the reference sequence B73v5 and the y-axis the -log10 of the p-value of associations of SNPs with F_v_/F_m_. Significance of associations of SNPs with F_v_/F_m_ (FDR < 15%) is indicated by symbols: empty circles (no significant association, ns); filled circles (significant association); triangle (SNP with highest significance, lead SNP, AX-90599221). Linkage disequilibrium (r^2^) between the lead SNP and SNPs in the target region is color-coded. QTL boundaries are given by vertical lines.

In the genome-wide association study (GWAS), significant marker-trait associations were detected for maximum quantum efficiency of photosystem II (PSII, F_v_/F_m_; Fig. 1A, Table 1) on chromosomes 1 (growth stage V6), 2 (V4, V6), 4 (V4), 8 (V4), and 10 (V4, V6). The QTL on chromosomes 1, 4 and 8 were detected in one growth stage only, while the QTL on chromosomes 2 and 10 were discovered in V4 and V6. Because the confidence intervals of the QTL on chromosomes 2 and 10 largely overlapped between the two growth stages, each genomic region was considered a single QTL affecting both growth stages in subsequent analyses. Together, the five QTL at the above-mentioned positions explained 50% and 59% of the genetic variance of F_v_/F_m_ in growth stages V4 and V6, respectively (Table 1). Here, we focus on the QTL on chromosome 10 spanning a 3.9 Mb genomic region at the distal end of the chromosome (Fig. 1). This QTL had a substantial effect size on F_v_/F_m_ of 0.45 (0.46) standard deviations (SD) and explained 35% (47%) of the genetic variance in V4 (V6). Due to a large effect in both growth stages, a biparental population was developed for fine-mapping and functional characterization of this region.

**Table 1:** QTL for maximum potential quantum yield of photosystem II (F_v_/F_m_) in landrace Kemater. The genome-wide association study (GWAS) was conducted using approximately 500,000 SNPs and adjusted entry means of F_v_/F_m_ in growth stages V4 and V6. Genomic positions are provided in B73v5 coordinates, and the number of genes within each QTL region is indicated. Effect SD: Absolute effect size from GEMMA output standardized by the standard deviation of the respective trait. GVE: Genetic variance explained by the respective QTL. Total GVE: Genetic variance explained by a model fitting all detected QTL in a growth stage simultaneously.

QTL	Growth stage	Chr	Start	End	Genes	Effect SD	GVE	Total GVE
qV4FvFm2.1	V4	2	22,392,853	25,329,743	79	-0.43	7%	50%
qV4FvFm4.1	V4	4	244,037,990	244,041,963	1	-0.32	7%	
qV4FvFm8.1	V4	8	40,669,624	40,670,607	1	1.35	5%	
qV4FvFm10.1	V4	10	148,413,861	152,303,594	194	-0.45	35%	
qV6FvFm1.1	V6	1	160,150,163	163,660,341	25	0.33	30%	59%
qV6FvFm2.1	V6	2	24,398,414	27,269,195	81	-0.39	12%	
qV6FvFm10.1	V6	10	148,506,993	152,303,594	192	-0.46	47%	

### Genetic dissection of the genomic region on chromosome 10

**Fig. 2:**
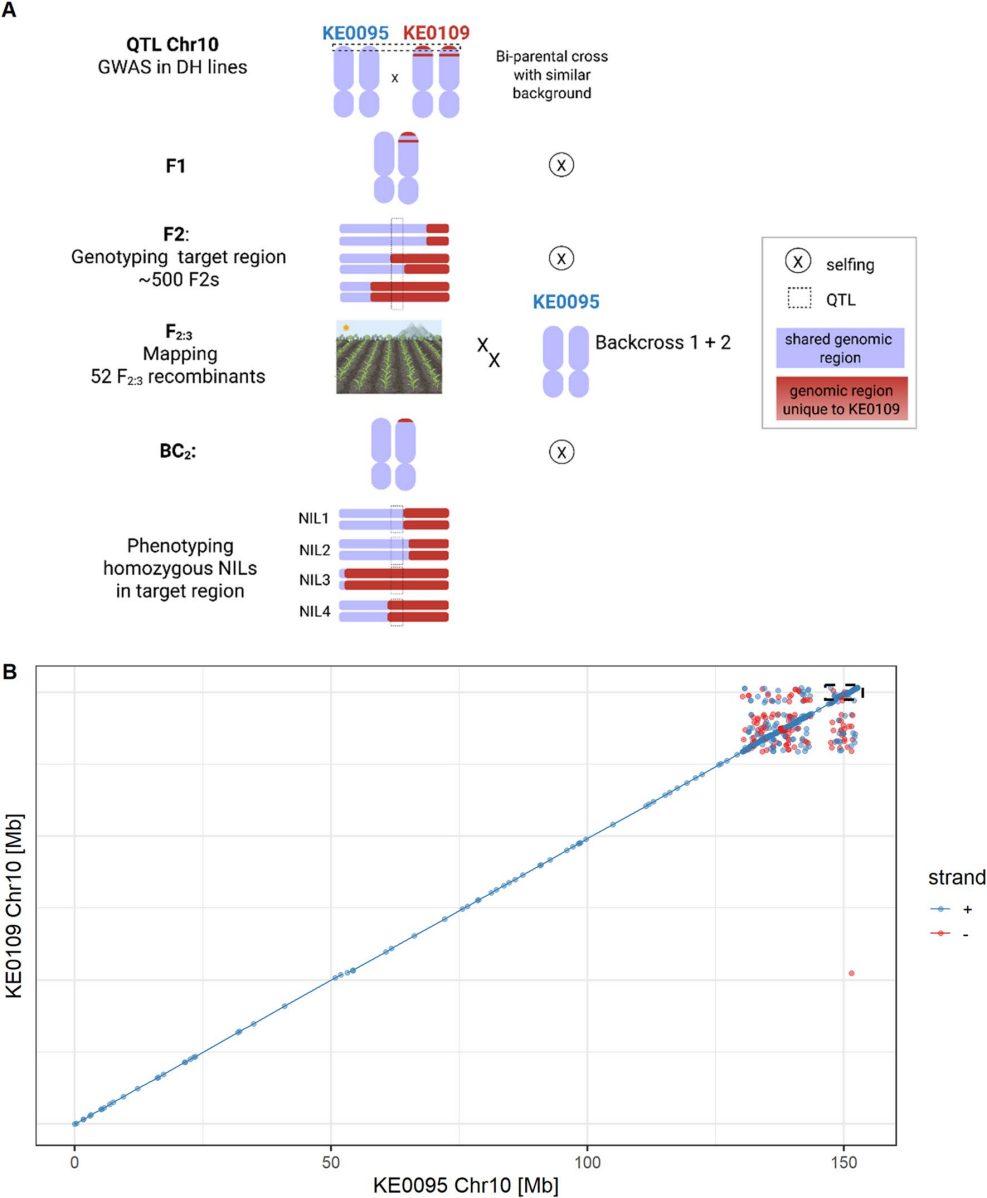
Material development for fine-mapping of a QTL for F_v_/F_m_ on chromosome 10. **A**) Schematic representation of the development of near isogenic lines segregating for a genomic region on chromosome 10 associated with F_v_/F_m_. Genomic fragments unique to the parental line KE0109 are highlighted in red. The QTL region is indicated by a dashed box. **B**) Pairwise sequence alignment of KE0095 and KE0109, the parents of the bi-parental population. The dashed box at the distal end of chromosome 10 marks the target region, which harbors the QTL for F_v_/F_m_. Alignments > 1 kb with sequence identity > 95% are shown: blue represents alignments on the forward strand, red indicates alignments on the reverse strand. Figure 2A was created with BioRender.com.

**Fig. 3:**
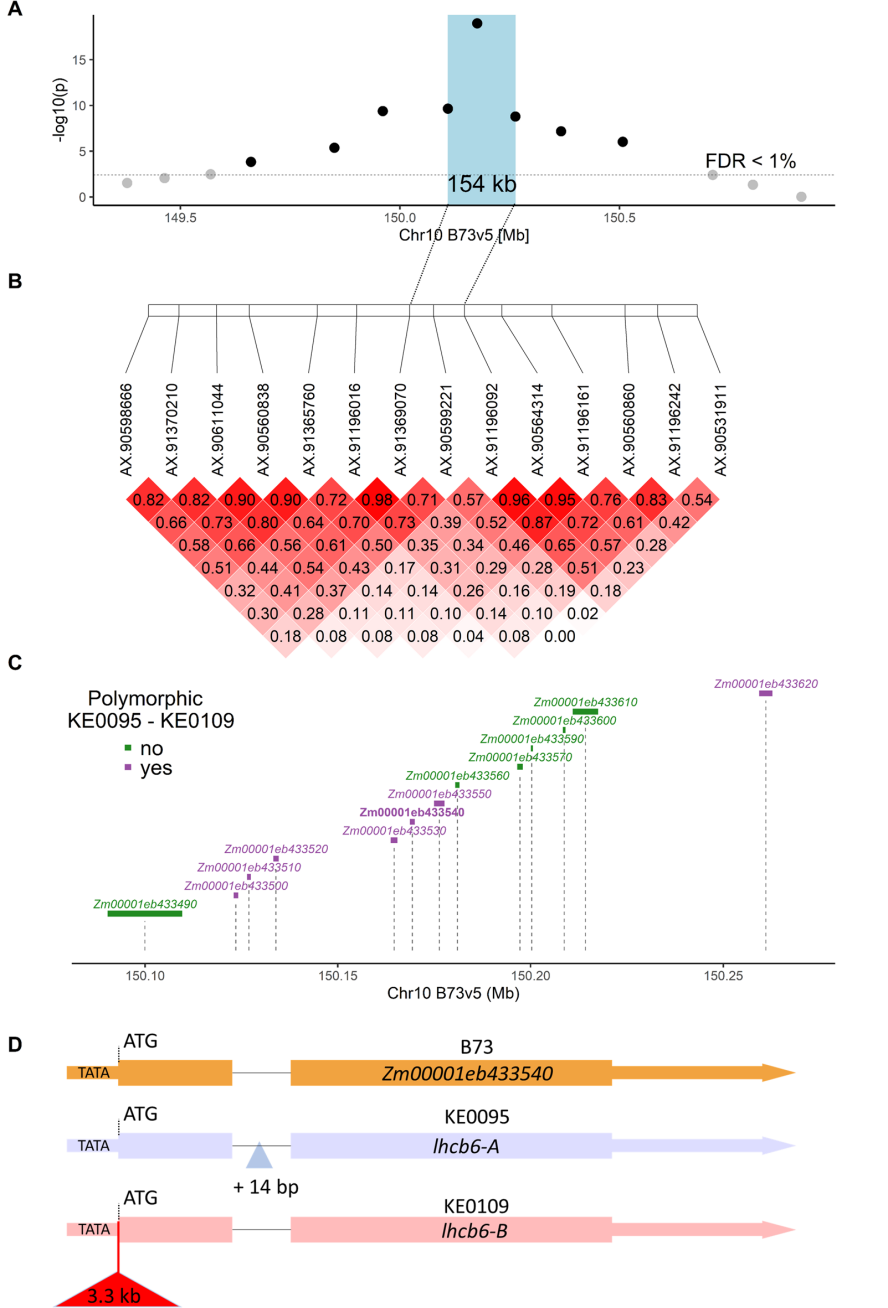
Characterization of a QTL region associated with F_v_/F_m_ in F_2:3_ recombinant lines. **A**) Association of 14 SNP markers with F_v_/F_m_ in F_2:3_ recombinant lines (*n* = 52) based on adjusted entry means across three field locations. The horizontal line indicates the false discovery rate (FDR) of 1%. The fine-mapped QTL region between the flanking markers of the marker with highest significance is indicated by a blue-shaded box. **B**) Linkage disequilibrium (r^2^) between 14 SNPs in the F_2:3_ recombinant lines. Dashed lines indicate the fine-mapped QTL region. **C**) Gene models located between the flanking markers of the fine-mapped QTL region. The size of the gene models is indicated by rectangles and their position on chromosome 10 in the reference sequence B73v5 is indicated by dashed vertical lines. Gene models with polymorphisms between KE0095 and KE0109 are shown in purple, and monomorphic gene models between KE0095 and KE0109 are shown in green. **D**) Gene models of the gene light harvesting chlorophyll a/b binding protein6 (lhcb6) in B73 (*Zm00001eb433540*), KE0095 and KE0109. Exons are indicated with rectangles, introns with a thin line and UTRs with a thick line. KE0109 carries a 3.3 kb genomic insertion immediately upstream of the start codon (red triangle). KE0095 carries a 14 bp insertion in the only intron of the gene.

Two Kemater DH lines, KE0095 and KE0109, were selected from the GWAS population as parents of a biparental population (Fig. 2A), based on their phenotypic difference in F_v_/F_m_ (Supplementary Figure S1) and their genomic similarity assessed with the 600k Axiom™ Maize SNP Array (600k Array)^29^. The two lines are polymorphic in the target region on chromosome 10 (Chr10:148,413,861−152,303,534; Fig. 1B), share the same SNP allele at the other four F_v_/F_m_ QTL (Table 1) and have a similar genomic background with 419,014 out of 493,989 SNPs being monomorphic (Supplementary Figure S2). Chromosome 10 is nearly identical between KE0095 and KE0109 based on sequence alignment (Fig. 2B) and 30,120 out of 32,236 SNPs being monomorphic. Polymorphic SNPs on chromosome 10 were confined to a 22 Mb region (Chr10:130,130,769–152,357,073), covering the QTL for F_v_/F_m_. Thus, the biparental population allowed fine-mapping of the target region (Chr10:148,413,861−152,303,534) with no interference of other QTL. F_2:3_ lines derived from the KE0095 x KE0109 cross, showing recombination events in the target region, were phenotyped for F_v_/F_m_ at the three German field locations Freising (FRS), Roggenstein (ROG) and Einbeck (EIN) in 2024. Entry-mean heritability (h^2^) for F_v_/F_m_ was 0.63 (95% CI: 0.40–0.77), indicating a significant genetic component underlying trait expression in the F_2:3_ recombinant lines. The association with F_v_/F_m_ was assessed for 14 markers in the target region, of which eight were significantly associated with F_v_/F_m_ (false discovery rate: FDR < 1%; Fig. 3A). The strongest association with F_v_/F_m_ was observed for the most significant SNP from the GWAS analysis (AX-90599221; lead SNP; Supplementary Table S1). The KE0095 allele of the lead SNP had a trait increasing effect on F_v_/F_m_ compared to the KE0109 allele, as expected from trait expression of the parents (Supplementary Figure S1). This marker explained 56% of the phenotypic and 98% of the genetic variance in the 52 F_2:3_ lines, suggesting that the underlying gene is the major determinant of F_v_/F_m_ segregation in this biparental population. The strength of the association with F_v_/F_m_ diminished by several orders of magnitude up- and downstream of the lead SNP (Fig. 3A). Recombination events were observed between the lead SNP and neighboring SNPs (AX-91369070, AX-91196092; maximum r^2^ = 0.71; Fig. 3B), allowing fine-mapping of the region. The fine-mapping narrowed the genomic region associated with F_v_/F_m_ to a 154 kb fragment between the markers AX-91369070 and AX-91196092 (Chr10:150,109,398−150,262,989; blue box in Fig. 3A, Supplementary Table S1).

### Molecular characterization of candidate genes

In the 154 kb region of interest, 13 genes are located in the B73v5 reference genome (Fig. 3C, Supplementary Table S2). Protein sequences of these genes were annotated and classified using MapMan4^30^, and proteins belonging to category “Photosynthesis” were selected. One gene met the selection criterion: *Zm00001eb433540 (light harvesting chlorophyll a/b binding protein6; lhcb6* also known as *CP24*). Since annotation alone reveals only genes with known functions, candidate gene prioritization was also conducted by comparing genomic sequences of KE0095 and KE0109.

Of the 13 genes in the target region, seven genes showed polymorphisms between KE0095 and KE0109, with five being non-synonymous leading to amino acid exchanges (Fig. 3C, Supplementary Table S3). Amino acid exchanges were non-conservative in two of these genes: *Zm00001eb433520* and *Zm00001eb433550*. The former encodes an uncharacterized protein and differs by multiple indels and four non-conservative amino acid exchanges between KE0095 and KE0109. The latter encodes PROTEIN S-ACYLTRANSFERASE38 and exhibits one non-conservative amino acid exchange (Supplementary Table S3). In addition, a 3.3 kb insertion directly upstream of the *lhcb6* translational start codon was detected in KE0109 which is not present in KE0095 and maize reference line B73 (Fig. 3D, File S1). The corresponding 5’ sequence of *lhcb6* in B73 and KE0095 contains a sequence resembling a TATA box (TATTATTATTAAT; Chr10:150,168,719−150,168,732, B73v5; Fig. 3D, Supplementary Figure S3) and is highly conserved across 33 maize genomes (Supplementary Figure S4).

**Fig. 4:**
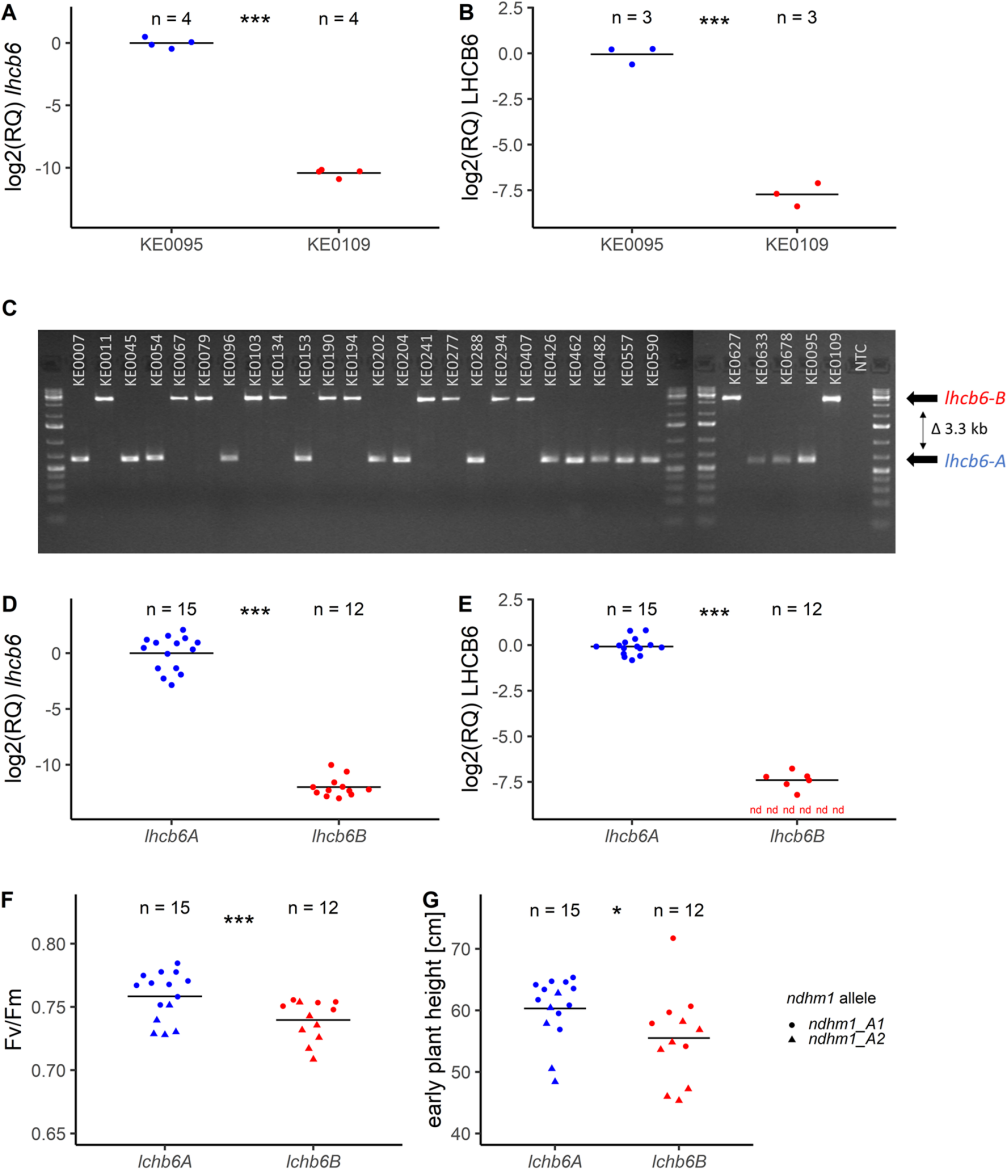
Molecular characterization of the candidate gene *lhcb6*. **A**,** B**) Log2-transformed relative transcript (**A**) and protein (**B**) levels of *lhcb6* in KE0095 and KE0109, normalized to KE0095. **C**) Genotyping of a 3.3 kb insertion in the genomic region upstream of the *lhcb6* start codon, assessed in a set of 27 Kemater lines and the parental lines KE0095 and KE0109. The lower band in the gel corresponds to the *lhcb6-A* allele and the higher band to the *lhcb6-B* allele, which contains the 3.3 kb genomic insertion. NTC: no template control **D-G**) Differences in transcript (**D**) and protein (**E**) abundance, F_v_/F_m_ **(F)** and early plant height **(G)** between 27 Kemater lines with contrasting *lhcb6* alleles. *lhcb6* (*lhcb6-A*, *n* = 15; *lhcb6-B*, *n* = 12) and *ndhm1* (*ndhm1-A1*, *n* = 15; *ndhm1-A2*, *n* = 12) segregate in the 27 Kemater lines. Genotypic data for *ndhm1* were derived from Urzinger et al.^18^. Significance was assessed by a two-way analysis of variance (ANOVA) including *lhcb6*, *ndhm1* and their interaction as factors. In **F** and **G** the *lhcb6* allele is indicated by color and *ndhm1* allele by shape of data points. Horizontal bars indicate group means and dots individual plant observations. Statistical differences were assessed by analyses of variance (ANOVA). ***p < 0.001; *p < 0.05. In **E** nd stands for not detected.

As a next step we investigated differences in transcript and protein abundance in leaves. In leaf proteomes of KE0095 and KE0109, 6,672 proteins were quantified. Of the 13 candidate genes two were represented (*Zm00001eb433540* [*lhcb6*], *Zm00001eb433610* [*Mg chelatase subunit H 1,chlh1*]; Supplementary Table S2). For *Zm00001eb433610* no difference in protein accumulation was observed between KE0095 and KE0109 (Supplementary Table S2), while protein accumulation of LHCB6 was reduced ~ 200 fold in KE0109 compared to KE0095 (Fig. 4B). The absence of the other 11 candidates in leaf proteomes of KE0095 and KE0109 cannot rule them out as candidate genes for the QTL but makes them less likely. For the two genes with non-synonymous amino acid exchanges (*Zm00001eb433520*, *Zm00001eb433550*) and *lhcb6*, transcript abundance was measured by RT-qPCR. No differences in transcript levels of *Zm00001eb433520* and *Zm00001eb433550* were observed between KE0095 and KE0109 (Supplementary Figure S5), while a 1000-fold reduction of transcript levels of *lhcb6* was detected in KE0109 compared to KE0095 (Fig. 4A).

Assuming that sequence variation at *lhcb6* determines differences in F_v_/F_m_, we further characterized variation at *lhcb6*. The 3.3 kb insertion is highly similar to a putative hAT transposon that is located approximately 600 kb downstream of *lhcb6* in B73 according to MaizeGDB (File S2, DTA_ZM00039). The insertion contains two long open reading frames (313 and 492 amino acids), encoding domains typically found in transposases—a TTF zinc finger domain, a Ribonuclease H-like superfamily domain, and a hAT C-terminal dimerization domain (InterPro accessions: IPR006580, IPR008906 and IPR012337). However, no terminal inverted repeats at the insertion site, which are characteristic for hAT transposons were detected in KE0109. Further, KE0095 contains a 14 bp insertion within the only intron of *lhcb6* compared to both B73 and KE0109 (Fig. 3D, File S1). Despite these differences in their DNA sequences, the amino acid sequences of LHCB6 in KE0095, KE0109, and B73 are identical. Hereafter, the KE0095 allele is referred to as *lhcb6-A* and the KE0109 allele with the 3.3 kb insertion is referred to as *lhcb6-B*.

Recently, *lhcb6* was proposed as a candidate gene for a QTL for F_v_/F_m_ in a maize MAGIC population, with maize line F7 carrying a deficient allele at this locus^20^. We compared the genomic sequences of F7, KE0095 and KE0109 and found that F7 carried an identical insertion in its putative promotor sequence of *lhcb6* as KE0109, corroborating an effect of the insertion on *lhcb6* transcript levels in a different genomic background.

To investigate if the variation in *lhcb6* transcript and protein levels is specific to KE0095 and KE0109, 27 Kemater lines were selected from the population of DH lines used in GWAS analyses for validation experiments. The DH lines were chosen to represent the two alleles of the lead SNP of the F_v_/F_m_ QTL on chromosome 10 in equal proportion. The presence or absence of the *lhcb6-B* allele was determined by PCR analysis, with primers spanning the putative transposon insertion. The *lhcb6-B* allele was detected in 12 of the 27 Kemater lines and was in complete linkage disequilibrium (LD) with the lead SNP from the GWAS in this set of Kemater lines (Fig. 4C, Supplementary Table S4). Corroborating results obtained with the two Kemater lines KE0095 and KE0109, RT-qPCR analysis revealed that *lhcb6* transcript levels were strongly decreased in the 12 Kemater lines carrying *lhcb6-B* compared to the 15 lines carrying *lhcb6-A* (Fig. 4D). The 27 Kemater lines under study also harbor different alleles at *ndhm1*, a gene which was shown to affect F_v_/F_m_ and early plant height in maize^18^. Correspondingly, of the 12 lines with *lhcb6-B*, LHCB6 protein could either not be detected (*n* = 6) or was strongly reduced (*n* = 6) compared to the 15 lines with *lhcb6-A* (Fig. 4E).

As expected from GWAS findings and results from F_2:3_ recombinant lines, mean F_v_/F_m_ was significantly (*p* < 0.01) decreased in DH lines with reduced LHCB6 protein levels in growth stage V6 (Fig. 4F, Supplementary Table S5). Further, early plant height in growth stage V6 was also significantly (*p* = 0.02) reduced in DH lines carrying the *lhcb6-B* allele (Fig. 4G, Supplementary Table S5). These results suggest that the transposon insertion in *lhcb6-B* diminishes *lhcb6* transcript abundance, leading to lower LHCB6 protein levels and thereby lower F_v_/F_m_.

To determine whether *lhcb6-B* is specific to the Flint maize heterotic group, haplotype diversity at this locus was investigated in 136 diverse inbred maize lines analyzed by Unterseer et al.^31^ and 501 DH lines derived from the Kemater landrace^32^. Haplotypes were defined by concatenating the 15 SNPs closest to *lhcb6* (8 upstream, 7 downstream). We identified 19 haplotypes with absolute counts ranging from 3 to 237 in the entire dataset. The most common haplotype (Hap1) had frequencies of 45% and 21% in the Kemater landrace and Flint breeding lines, respectively, but was absent in elite Dent lines (Supplementary Figure S6). Based on the results of PCR genotyping, Hap1 was in complete LD with the insertion in *lhcb6-B* in the subset of 27 Kemater lines (Supplementary Table S4). These findings can be extended from the landrace population, provided that the association between Hap1 and *lhcb6-B* is consistent in breeding lines.

### Functional characterization of the *lhcb6-B* allele

**Fig. 5:**
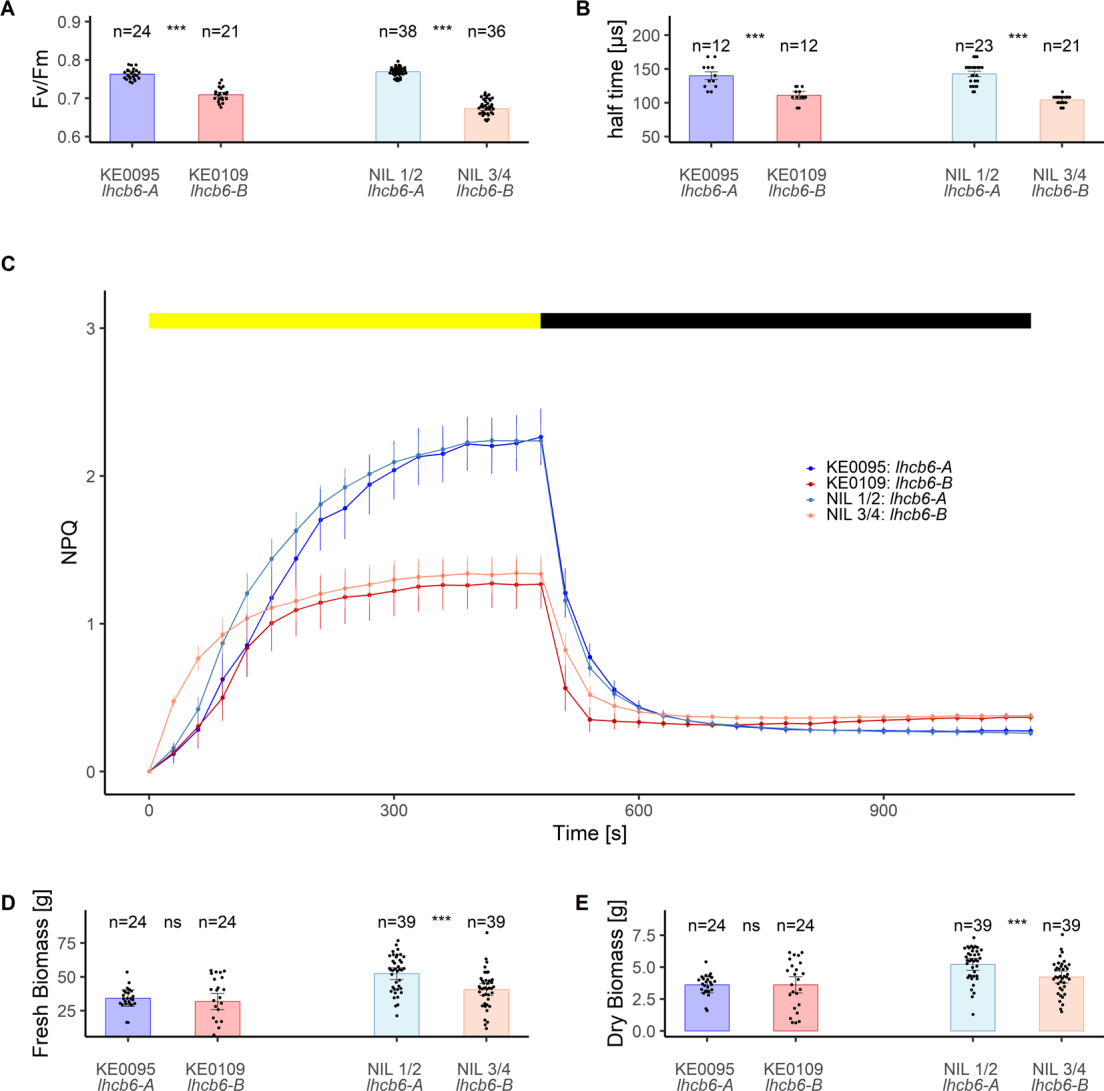
Physiological and agronomic evaluation of near isogenic lines (NILs) with contrasting *lhcb6* alleles and of parental lines KE0095 and KE0109. Plants were grown in a growth chamber under fluctuating light intensity until growth stage V6. **A**) Maximum quantum yield of photosystem II (F_v_/F_m_). **B**) Functional antenna size estimation based on the half-time to the J level (T₁/₂) during an OJIP fluorescence induction curve. **C**) Non-photochemical quenching (NPQ) dynamics during an eight-minute light phase (yellow bar; actinic light intensity: 1500 µmol m^−2^s^− 1^) followed by a ten-minute dark phase (black bar). Dots represent adjusted means, bars represent standard errors (SE; KE0095: n = 4, KE0109: n = 4, NIL1/2: n = 8, NIL3/4: n = 7 ). **D)** Fresh biomass. **E**) Dry biomass. Measurements were taken at growth stages V5–V6. Significant differences based on Student’s t-tests are indicated by stars. ***p < 0.001; ns: not significant. Bars represent adjusted means ± SE and dots represent individual plant observations (**A**, **B**, **D**, **E**).

To evaluate the effect of the *lhcb6-A* and *lhcb6-B* alleles on phenotypic traits other than F_v_/F_m_, we developed four near isogenic lines (NILs) with contrasting *lhcb6* alleles from the F_2:3_ recombinant lines. NIL 1 and NIL 2 differ from NIL 3 and NIL 4 in a 154 kb genomic fragment harboring *lhcb6* and 12 other genes defined by fine-mapping in F_2:3_ recombinant lines (Fig. 3). NIL 1 and NIL 2 carry *lhcb6-A*, while NIL 3 and NIL 4 carry *lhcb6*-*B* (Supplementary Figure S7, Supplementary Table S6). In addition to F_v_/F_m_, we measured the effective PSII antenna size, NPQ, and fresh and dry biomass of the NILs. As expected, F_v_/F_m_ was lower in NILs carrying *lhcb6-B* compared to NILs carrying *lhcb6-A* (Fig. 5A), which was the result of a combination of higher minimal fluorescence (F_0_; Supplementary Figure S8A) and lower maximal fluorescence (F_m_; Supplementary Figure S8B). Effective antenna size of PSII was determined from the half -time needed to reach the J level of the initial O-J rise in an OJIP chlorophyll induction curve^33^. The half-time was significantly lower in KE0109 and NILs carrying the *lhcb6-B* allele indicating larger effective antenna size (Fig. 5B). During the light period, maximum NPQ in NILs with *lhcb6-B* and KE0109 was approximately half of that observed in NILs with *lhcb6-A* and KE0095 at the given actinic light intensity (Fig. 5C). Conversely, NPQ values at the end of the dark period were increased in NILs with *lhcb6-B* and KE0109 (Fig. 5C), suggesting impaired relaxation of NPQ. Fresh and dry biomass were reduced in NILs with *lhcb6-B* compared to NILs with *lhcb6-A*, but this reduction was not observed in KE0109 compared to KE0095 (Fig. 5D, E).

**Fig. 6:**
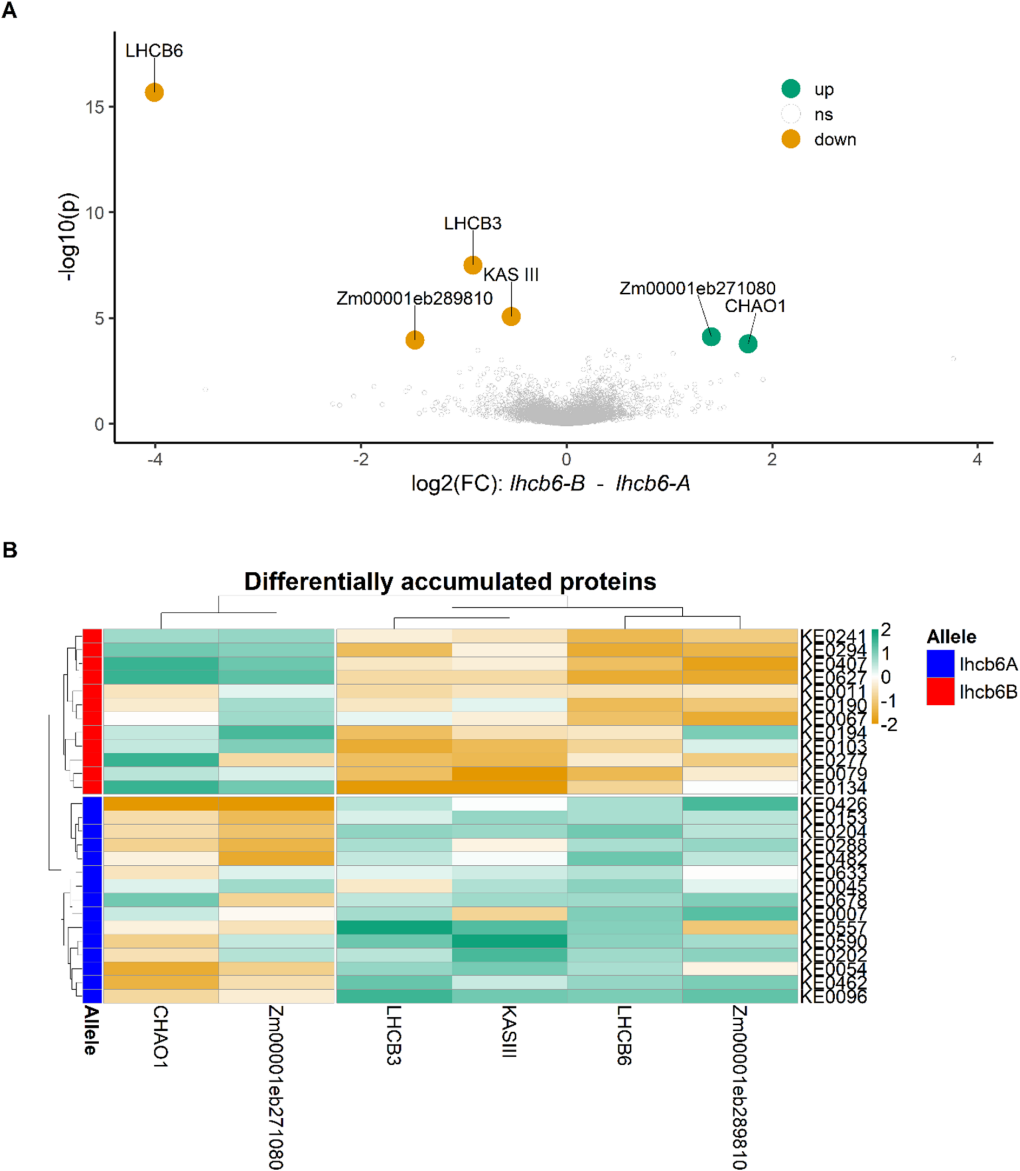
Leaf proteome analysis of 27 Kemater lines segregating for *lchb6*. **A**) Differentially accumulated proteins in 27 Kemater lines grouped by their *lhcb6* allele. Proteins were considered differentially accumulated at a false discovery rate (FDR) < 20%. Proteins with lower abundance in lines carrying the *lhcb6-B* allele are shown in orange, while those with higher abundance are shown in green. **B**) Relative quantities of six significantly differentially accumulated proteins from (**A**) expressed as z-scores for each of the 27 Kemater lines. Lines carrying the *lhcb6-A* allele are indicated by the blue and those with the *lhcb6-B* allele with the red bar.

We further explored the consequences of reduced *lhcb6* expression on PSII light-harvesting complex (LHCII) antenna components in the subset of 27 Kemater lines described earlier. Based on sequence similarity to experimentally annotated Arabidopsis (*Arabidopsis thaliana*) LHCII components, 13 putative LHCII subunits were detected in the maize reference line B73 (Supplementary Table S7). One of these is an uncharacterized paralog of LHCB6 (Zm00001eb066480, LHCB6-2). LHCB6-2 has an amino acid sequence identity of 93% (235/253 amino acids) with LHCB6-1, including a four amino acid deletion at the N-terminus in LHCB6-2 compared to LHCB6-1. Of these 13 proteins, 12 were detected and quantified in the leaf proteomes of the 27 Kemater lines, including the three major LHCII antenna proteins (LHCB1, LHCB2 and LHCB3) and the three minor LHCII antenna proteins (LHCB4, LHCB5 and LHCB6). In addition to the expected reduction in LHCB6, LHCB3 (Zm00001eb324240) protein levels were decreased by ~ 50% in *lhcb6-B* lines. The abundance of the other 10 quantified LHCII proteins, including LHCB6-2, remained unchanged. We also analyzed differential accumulation among all 5,531 quantified proteins. Only six proteins exhibited differential accumulation based on *lhcb6* allele status (Supplementary Table S8, Fig. 6). The *lhcb6-B* allele was correlated with a reduced abundance of four proteins and an increased abundance of two proteins (Fig. 6, Supplementary Table S8). Notably, LHCB6 showed the most significant reduction, followed by LHCB3 (Fig. 6A).

Of the two other proteins with decreased accumulation in Kemater lines with *lhcb6-B* (Zm00001eb068760, Zm00001eb289810), only one has a clearly defined function: a 3-oxoacyl-[acyl-carrier-protein]-synthase III (KAS III; Zm00001eb068760). KAS III catalyzes the first step in the fatty acid synthesis pathway II, which is essential for the synthesis of membrane phospholipid acyl chains^34^.

Among proteins with increased abundance, CHLOROPHYLLIDE A OXYGENASE1 (CAO1; Zm00001eb039350) showed the strongest upregulation—a threefold increase in *lhcb6-B* lines (Fig. 6, Supplementary Table S8). Another protein with increased accumulation is of unknown function (Zm00001eb271080).

## Discussion

A profound understanding of the genetic basis of photosynthetic efficiency can leverage the improvement of agricultural productivity^9,24,35^. Previous research has established a genetic correlation between the maximum quantum yield of photosystem II (PSII, F_v_/F_m_) and agronomically relevant traits like early development and chilling stress tolerance in maize, and links it to other photosynthetic traits, including PSII operating efficiency (ΦPSII), non-photochemical quenching (NPQ), and chlorophyll content^11,18,20,21^. QTL with large effects on F_v_/F_m_ have been mapped in maize and several candidate genes have been proposed, but their underlying genetic mechanisms have remained largely unknown^20,25–28,36^.

In this study, we identified five QTL for F_v_/F_m_ across two early growth stages segregating in a maize landrace. The causal gene underlying the QTL on chromosome 2 (*ndhm1*) with pleiotropic effects on early plant growth and F_v_/F_m_ has been characterized by our group recently and encodes a subunit of the NADH-dehydrogenase like (NDH) complex^18^. Here, we could show that *lhcb6* (also known as *CP24*), a minor antenna component of the light-harvesting complex II (LHCII), is the causal factor underpinning the major QTL for F_v_/F_m_ on chromosome 10. Both *lhcb6* and *ndhm1* play key roles in dynamic regulation of photosynthesis, by balancing photoprotection and efficiency via NPQ and cyclic electron transport, respectively^37,38^. We therefore conclude that F_v_/F_m_ can serve as a proxy trait for identifying meaningful genetic variation that contributes to photosynthetic regulation in changing environmental conditions.

In a recent publication, Ferguson et al.^20^ proposed *lhcb6* as candidate gene for a QTL affecting F_v_/F_m_, ΦPSII and NPQ in a maize multi-parent advanced generation inter-cross (MAGIC) population. They showed that a specific haplotype found in the Flint line F7 was associated with reduced transcript and protein levels of *lhcb6* compared to the maize reference line B73. Our current study detected a QTL in the same genomic region in a Flint maize landrace. Ferguson et al.^20^ based their evidence for *lhcb6* being the causal gene on functional annotation, physiological similarity to Arabidopsis mutants, and expression differences between B73 and F7, leading to impaired assembly of higher order PSII-LHCII supercomplexes. By mapping short-read sequencing data from F7 against the B73 reference, they identified three SNPs and one indel specific to the F7 haplotype altering computationally predicted transcription factor binding sites (TFBS). Here, we used a forward genetic approach with near isogenic lines (NILs) derived from a maize landrace in combination with long-read genome assemblies of their two parental lines KE0095 and KE0109 and we could pinpoint the causal polymorphism underlying this QTL to a 3.3 kb insertion of a hAT transposon directly upstream of the start codon of *lhcb6* in the *lhcb6-B* allele. The functional importance of the affected region as promotor of *lhcb6* is supported by its conservation across a diverse panel of maize lines, its overlap with a region of accessible chromatin, indicating its accessibility to transcription factors^39^, computationally predicted TFBS^20^, and a putative TATA-box. The insertion of a hAT transposon in *lhcb6-B* shifts the putative promotor region of *lhcb6* 3.3 kb upstream, extending the typical distance of *cis-*regulatory elements to the transcription start site in maize^40^. We identified the same mutation in the Flint line F7, linking our findings with those of Ferguson et al.^20^. Thus, our results strongly support the hypothesis that *cis*-genic allelic variation at *lhcb6* is causative for differential gene expression, leading to significantly reduced RNA and protein levels, and further enabled us to identify the specific underlying structural variation.

In most vascular plants, the LHCII antenna consists of major antennae (homo- or heterotrimers of LHCB1, LHCB2, and LHCB3) and minor antennae (monomers of LHCB4, LHCB5, and LHCB6)^41,42^. These antennae assemble into PSII supercomplexes (e.g., C_2_S_2_M_1_, C_2_S_2_M_2_, C_2_S_2_), which are composed of two core (C) subunits of PSII, two strongly (S) bound trimers, and zero, one, or two moderately (M) bound trimers. In these supercomplexes, the M trimer is coordinated by LHCB3 and LHCB6^43^. While nearly all mechanistic insights into LHCII organization and function have come from studies on *Arabidopsis thaliana* mutants it has remained unclear whether the proposed principles hold in a C_4_ crop with different photosynthetic architecture. Here, we provide insights into LHCII function and its natural variation in maize. Our proteome analyses show both significantly reduced LHCB6 and LHCB3 levels in DH lines carrying the *lhcb6-B* allele reflecting the direct interaction between the two proteins. In Arabidopsis, a lack of LHCB6 protein impairs LHCII assembly, favoring the formation of C_2_S_2_ complexes^44^. Conversely, we observed that estimates of the functional antenna size based on chlorophyll fluorescence induction curves were increased in NILs carrying the *lhcb6-B* allele and KE0109, matching observations in F7^20^ and *lhcb6* and *lhcb6 lhcb3* double mutants of Arabidopsis^45^. Ilíková et al.^45^ proposed that despite the changes in LHCII assembly, the M-trimers remain functionally connected to PSII arrays in the *lhcb6* mutants, explaining the increased functional antenna size. Further, they suggest that the unbound domains of LHCII are responsible for increased F_0_ values observed in the mutants. In accordance with these findings, we also observed no change in the abundance of light harvesting proteins (Supplementary Table S7) other than LHCB6 and LHCB3 in the subset of 27 Kemater lines and increased F_0_ in NILs carrying *lhcb6-B* and in parent KE0109.

Ferguson et al.^20^ proposed that *lhcb6-B* might be specific for the Flint heterotic group. Here, we could show that, despite its negative effect on LHCB6 abundance, it is present at high frequency in Flint breeding lines and is absent in Dent lines with publicly available genomic information. Whether the Flint specificity of *lhcb6-B* reflects beneficial effects of this allele in temperate climates or has arisen solely from random genetic drift has remained unclear and warrants further investigation.

LHCII plays a dual role in light harvesting and photoprotection. It captures and transfers light energy to PSII and is involved in photoprotection through NPQ, a mechanism that dissipates excess light energy as heat^37^. Genetic variation for NPQ has been reported for maize and sorghum (*Sorghum bicolor*) populations^46,47^. In maize, LHCII regulation responds to light and temperature^48^, with implications for state transitions and energy balance between the two photosystems^49,50^. Thus, targeting LHCII dynamics is a potential strategy to enhance stress resilience^51^ and maize landraces can enhance our understanding of these complex physiological processes^9,13^.

Interestingly, although *lhcb6* mutants severely impair growth in Arabidopsis^44,52,53^, the *lhcb6-B* allele in maize causes only mild growth reductions, suggesting compensatory mechanisms in the genome. To explore this hypothesis, we analyzed genomic and proteomic data in our landrace population with the aim of identifying factors that attenuate the effects of *lhcb6-B* in maize. Our analysis revealed a previously uncharacterized paralog of LHCB6 in maize, which we designated as LHCB6-2 (Zm00001eb066480). While paralogs for LHCB1, LHCB2, and LHCB4 have been reported in Arabidopsis^54^, this is, to our knowledge, the first description of a LHCB6 paralog. The two maize LHCB6 paralogs are highly similar (File S3), with most differences occurring at the N-terminus, a region that includes a potential phosphorylation site^42^. Although phosphorylation is known to regulate LHCB proteins in species such as spinach (*Spinacia oleracea*), Arabidopsis, and *Chlamydomonas reinhardtii*, it has not been documented for LHCB6^55,56^. As LHCB6-2 cannot compensate for the deficiency of LHCB6-1 (Zm00001eb433540) in the Kemater DH lines, we propose that LHCB6-1 and LHCB6-2 may have distinct roles. While LHCB6-1 is important for photoprotection balanced by NPQ, LHCB6-2 may primarily facilitate efficient light capture for photosynthesis, partially compensating for negative effects on biomass accumulation. However, this hypothesis requires further analyses which are beyond the scope of this study.

Comparing the leaf proteome between contrasting Kemater lines carrying either the *lhcb6-A* or the *lhcb6-B* alleles, we observed co-expression of several proteins shedding light on the dynamic regulation of the LHCII antenna. For instance, CHLOROPHYLLIDE A OXYGENASE1 (CAO1; also known as CHAO1), which converts chlorophyll a to chlorophyll b and is essential for LHCII antenna assembly^57^, shows elevated levels in lines carrying *lhcb6-B*. Increased CAO1 may serve as a compensatory feedback mechanism in response to LHCII antenna deficiency. A connection between chlorophyll b levels and LHCII efficiency has been shown in other species. In Arabidopsis and barley (*Hordeum vulgare*), deficiencies in chlorophyll b have been shown to lead to reduced accumulation of LHCII components^58,59^, and overexpression of CAO1 in Arabidopsis enlarged the LHCII antenna^60^. Further, an uncharacterized protein potentially involved in lipid transfer (Zm00001eb271080) has been linked to variation in maize tocopherol and tocotrienol content^61^. Given the protective role of tocopherols against photoinhibition and lipid photooxidation^62^, its increased protein levels might indicate increased oxidative stress associated with impaired NPQ, similar to the increased reactive oxygen species (ROS) observed in Arabidopsis *lhcb6* mutants^63^. Reduced protein levels of KAS III, an enzyme involved in lipid biosynthesis, may reflect disturbances in the assembly of the PSII–LHCII supercomplex, where lipids play a stabilizing role^64^.

Our findings highlight the value of natural genetic variation for unraveling the complex regulation of photosynthesis and for enhancing crop performance under stress conditions with significant implications for breeding. The development of a diagnostic marker capturing the allelic state of *lhcb6*—including the hAT transposon insertion in the promotor—and using this marker alone or in combination with two or more flanking markers to define a diagnostic haplotype will facilitate the evaluation of the gene’s effect in segregating populations and enable any type of marker-based selection including whole-genome selection. In addition, the screening of additional natural genetic variation and the generation of new variation at *lhcb6* via chemical induced mutations, genome editing, or classical transgenic approaches may allow engineering of the LHCII antenna and modification of NPQ in breeding material. Beneficial alleles detected or created in this manner can be introgressed into elite material through marker assisted backcrossing.

## Materials and methods

### Plant Material

The landrace “Kemater Landmais Gelb” (Kemater) was chosen as the base population for this study due to its phenotypic variation for early development, as well as its fast decay of linkage disequilibrium (LD) and absence of pronounced population structure. Kemater was selected from a set of 35 European maize landraces covering a broad geographical region described by Mayer et al.^65^. Kemater represents 77% of the molecular variance of the entire collection^11^ based on the 600k Axiom™ Maize Genotyping Array (600k Array)^29^. Directly from the landrace, 516 doubled-haploid (DH) lines were produced and multiplied using the in vivo haploid induction method^66^, and 222 (growth stage V4) and 211 (V6) DH lines were phenotyped for F_v_/F_m_ in 2017 and 2018. A curated dataset comprising 501 Kemater DH lines genotyped with 501,124 markers from the 600k Array was obtained from Mayer et al.^32^.

A subset of 27 DH lines was selected from the full set of Kemater lines for functional validation of a QTL for F_v_/F_m_ on chromosome 10 (Chr10:148,413,861−152,303,534; Table 1, Fig. 1), including phenotypic, transcript and proteome measurements. These lines were chosen to obtain a balanced frequency of two contrasting alleles at the QTL (validation set; Supplementary Table S4).

All plant-related experiments were conducted in accordance with the internal guidelines of the Technical University of Munich as well as all applicable national and international regulations. Compliance with the Nagoya Protocol for all material derived from the landrace Kemater was ensured through standard material transfer agreements.

### Development of recombinant and near isogenic lines

A schematic overview of the material development for fine-mapping is presented in Fig. 2A. Two Kemater DH lines, KE0095 and KE0109, were chosen for their contrasting alleles at the QTL for F_v_/F_m_ on chromosome 10, while being as similar as possible in their genomic background (Fig. 2B, Supplementary Figure S2) and crossed to produce a biparental population. KE0095 and KE0109 are polymorphic for 74,975 of 493,989 markers of the 600k Array^29^. KASP markers polymorphic between the two parental lines and positioned in the QTL region on chromosome 10 were synthesized using probe sequences of the 600k Array. F_2_ lines were genotyped with six KASP markers and individual plants showing recombination between markers AX-90560736 (Chr10:143,112,717; Supplementary Table S1) and AX-91196698 (Chr10:152,357,073; Supplementary Table S1) were self-pollinated. Resulting F_2:3_ recombinant lines were genotyped with 14 KASP markers (Supplementary Table S1) and phenotyped for F_v_/F_m_ in field experiments at three German locations in 2024. Selected F_2:3_ recombinants were backcrossed two times to KE0095 as the recurrent parent. BC_2_ was genotyped with a 15k SNP chip to select individual plants with minimum donor parent genome. These were selfed to obtain four near isogenic lines (NILs) which were phenotyped in growth chamber experiments.

### Field experiments

The Kemater DH lines were evaluated in 2017 and 2018 as part of a broader study on landrace diversity^11^. Briefly, in two locations, Golada and Tomeza, 222 Kemater DH lines were phenotyped for F_v_/F_m_. The experimental design was a 10 × 10 lattice design with two replications at each site. As checks, fourteen Flint and one Dent inbred line as well as the original landrace population (each replicated four times) were used. Further information on field trial locations, check varieties, and methods for phenotypic data analysis is available in the publications of Hölker et al.^11^ and Mayer et al.^32^.

Phenotypic evaluation of the set of 27 Kemater DH lines and the population of recombinant F_2:3_ lines used for fine-mapping was conducted in Einbeck (EIN, Germany, 51°49’05.9"N 9°52’00.3"E), Roggenstein (ROG, Germany, 48°10’47.5"N 11°19’12.9"E), Bernburg (BBG, Germany, 51°49’28.6"N 11°42’26.3"E) and Freising (FRS, Germany, 48°24’13.2"N 11°43’28.5"E). The subset of 27 Kemater DH lines was grown in a randomized complete block design (RCBD) with two replications in locations ROG, FRS and Oberer Lindenhof (OLI, Germany, 48°28’26.3"N 9°18’17.9"E) in 2023 together with four inbred checks. The 52 F_2:3_ recombinant lines were evaluated in a RCBD with two replications in EIN, ROG and FRS in 2024 using the same inbred checks. Plots consisted of 20 plants grown in single rows of 3 m length with 0.75 m spacing between them (9 plants/m^2^). Field trials were subjected to standard agricultural practices. Phenotypic observations from plots containing at least five plants were filtered by a Grubbs’ outlier test^67^.

### Growth conditions in growth chamber experiments

For phenotyping of the four NILs, alongside their recurrent parent (KE0095) and donor parent (KE0109) in growth chamber experiments, kernels were immersed in water for 5 min, placed on filter paper and allowed to pre-germinate in the dark at 28 °C for 72 h. After germination, samples from individual seedlings were collected for DNA extraction and the seedlings were transplanted to small pots filled with CL ED73 soil (Einheitserdewerke Patzer, Germany) as described in Urzinger et al.^18^. Plants were grown in growth chambers with 16/8 h day night [d/n], 25/20°C d/n, 75% relative humidity [RH] until growth stage V4 to V6. To simulate fluctuations in light intensity occurring in the field, a light switch scheme was employed, alternating between 30 min high light (800 µmol m^−2^s^− 1^ photosynthetically active radiation [PAR]) and 15 min low light (100 µmol m^−2^s^− 1^ PAR). The four NILs and the two parental DH lines KE0095 and KE0109 were replicated 10 times in each of the two blocks in a RCBD. The developmental stages of maize were determined according to the leaf collar method^68^. Phenotypic data from growth chamber experiments were filtered by a Grubbs’ outlier test^67^.

### Measurement of photosynthetic traits

F_v_/F_m_ was measured in growth chamber grown plants using a LI-600 (LI-COR Inc., Lincoln, NE, USA). Measurements were taken pre-dawn on the last fully developed leaf of plants that had been dark-adapted overnight. In field experiments, the last fully developed leaf was cut from the plant and dark-adapted for at least 30 min before measuring. F_v_/F_m_ was assessed at growth stage V6 with a LI-6800 Portable Photosynthesis System (LI-6800; LI-COR Inc., Lincoln, NE, USA) as described in Urzinger et al.^18^. The last fully developed leaf was clipped at the midpoint, avoiding the midvein. Chamber flow rate was set to high (150 µmol s⁻¹) with a match frequency of 10. Maximum fluorescence (F_m_) of dark-adapted leaves was obtained using a rectangular saturating flash (6000 µmol m⁻² s⁻¹, 800 ms) with fluorescence acquisition at a modulation rate of 5 Hz. Fluorescence constants used for electron transport calculations were set to a leaf light absorptance of 0.8 and a fraction of absorbed light partitioned to PSII of 0.5. Non-photochemical quenching (NPQ) was measured in growth chamber grown plants using a LI-6800. Measurements were taken pre-dawn on the last fully developed leaf of plants that had been dark-adapted overnight. Dark-adapted maximum fluorescence (F_m_) was determined using a rectangular saturating flash (6000 µmol m⁻² s⁻¹, 800 ms) with an output rate of 100 Hz, margin of 5 points and dark mode rate of 50 Hz. Light-adapted maximum fluorescence F_m_` was determined after setting the actinic light to 1500 µmol m^−2^s^− 1^ by conducting 16 saturating flashes every 30 s from 30 s up to 480 s after illumination and after subsequently switching off the actinic light by another set of 20 saturating flashes every 30 s from 30 s to 600 s after dark. NPQ was calculated for each measurement timepoint separately as (F_m_ - F_m_`) / F_m_`. For estimation of PSII antenna size, fast chlorophyll a fluorescence induction kinetics (OJIP transients) were measured by a flash with intensity of 20,000 µmol m^−2^s^− 1^ and a duration of 600 ms with a LI-6800. The Dark mode rate was set to 500 Hz, the Light mode rate to 1 kHz and the Flash mode rate to 250 kHz. The half-rise time t_1⁄2_ from the minimal fluorescence F_0_ to the fluorescence at time point J (F_j_) was used as an estimate of the effective antenna size of PSII^33^.

### Plant height measurement in the field

Early plant height was measured by stretching all leaves of a plant to measure the maximum length between soil and the tip of the leaves in growth stages V4 and V6. The measurements of three individual plants per plot were averaged to obtain the plot level measurement.

### Identification of trait associations

We conducted a genome-wide association study (GWAS) for F_v_/F_m_ in growth stages V4 and V6 in the Kemater population with the software GEMMA (v 0.98.1)^69^. The same population and phenotypic data as reported in Urzinger et al.^18^ were used. Genotypic data for the 222 Kemater DH lines was obtained from Hölker et al.^70^ and filtered for polymorphic SNPs. Phenotypic data for F_v_/F_m_ estimates were obtained from Hölker et al.^11^. Briefly, F_v_/F_m_ was assessed in a set of Kemater DH lines (V4: *n* = 222; V6: *n* = 211) using a fluorometer (OS-30p, Opti-Sciences Inc., USA) at growth stages V4 in both 2017 and 2018, and V6 in 2017. The following statistical model was used:

$$\boldsymbol{y}=1\alpha+\mathbf{x}\beta+\mathbf{Z}\mathbf{u}+\mathbf{e}$$

where **y** is the *n*-dimensional vector of adjusted entry means for F_v_/F_m_ averaged across four (V4) and two (V6) environments respectively, with *n* being the number of DH lines; α is the intercept; *β* is the fixed effect of the tested SNP; **x** is the vector of corresponding genotype scores coded as 0 or 2; **u** is the *n*-dimensional vector of random genotypic effects, with **u** ~ *N*(**0**,**K***σ*^*2*^_*g*_); and **e** is the *n*-dimensional vector of random residual effects, with **e** ~ *N*(**0**,**I**_*n*_*σ*^*2*^). **K** denotes the (*n* × *n*) genomic kinship matrix based on SNP markers, calculated with GEMMA (“-gk-2”). **I**_*n*_ denotes the (*n* × *n*) identity matrix. *σ*^*2*^_*g*_ and *σ*^*2*^ refer to the genetic and residual variance pertaining to the model, respectively. The matrix **Z** (*n* × *n*) assigns adjusted means to the random genotypic effects. Significance of trait associations was assessed with a likelihood ratio test, as implemented in GEMMA, using a 15% false discovery rate (FDR)^71^. Pairwise r^2^ between SNPs was calculated as their squared pearson correlation coefficient^72^. SNPs with an r^2^ ≥ 0.6 and within a physical distance < 1 Mb were considered to belong to the same QTL. Physically overlapping QTL were merged and the resulting QTL was described by the start and end position of the first and last SNP from the merged QTL. The proportion of genetic variance explained by a single QTL was estimated by calculating the reduction in genetic variance between a full model including all QTL and a reduced model excluding the respective QTL, following Mayer et al.^12^.

### Transcript level measurements

RNA was extracted from leaves using a guanidine hydrochloride protocol^73^, followed by DNase digestion and first-strand cDNA synthesis (Maxima H Minus Kit, random hexamer primers, Thermo Scientific K1652). RT-qPCRs were performed in technical triplicates with both primers binding to the 3’ UTR of *lhcb6*,* Zm00001eb433520 and Zm00001eb433550* (Supplementary Table S9). For normalization, RT-qPCR results of the house-keeping gene MEP served as reference (membrane protein PB1A10.07c, *Zm00001eb257640*).

### Leaf proteome analysis

For proteome measurements the parental lines KE0095 and KE0109 were grown in a growth chamber as described above. Leaf material was sampled in frozen nitrogen for three biological replicates per genotype in growth stage V4. From the subset of 27 DH lines grown in field experiments, a pooled sample of six plants from two replicates of one genotype was taken from the last fully developed leaf in growth stage V6 and frozen in liquid nitrogen. Total proteome was extracted and measured following an established protocol^74^. For quantification of peptides an additional labeling step was added after protein digestion. Samples of KE0095 and KE0109 were labeled using 16-plex tandem mass tag (TMT) reagent, while samples from the subset of 27 DH lines were labeled using 11-plex TMT reagent and processed in three batches. A detailed protocol for sample preparation and mass spectrometry was described by Urzinger et al.^18^. For normalization of batch effects in the subset of 27 Kemater lines, a reference was prepared by mixing equal peptide amounts of all samples which was measured twice in each of the three TMT batches. Proteins were identified using the proteome of B73v5 as reference^75^ and quantified by MaxQuant^76^. To avoid imputation of missing values in quantitative analyses, only proteins which were identified in all samples of an experiment were considered. Log2 transformed raw intensities of identified proteins were median normalized to remove differences in summed intensities per sample. For the subset of 27 Kemater lines the two reference channels per TMT batch were used to calculate a correction factor for each identified protein per TMT batch.

### Detection of the *lhcb6-B* allele

To determine presence or absence of a 3.3 kb insertion in the genomic sequence upstream of the *lhcb6* start codon (Fig. 3D) a PCR assay was developed. Primer sequences are listed in Supplementary Table S9. PCR reactions were performed using Q5 High-Fidelity DNA Polymerase (New England Biolabs Inc., Ipswich, MA, USA) according to the manufacturer’s specifications at an annealing temperature of 65 °C for 15 s followed by elongation at 72 °C for 30 s. Amplicon lengths of genomic sequences were discriminated by gel electrophoresis of PCR products. Sanger sequencing of PCR amplicons was performed using Mix2seq kits at Eurofins Genomics Germany GmbH (Ebersberg, Germany).

### Whole-genome sequencing

DH lines KE0095 and KE0109, from which recombinant lines and NILs were derived, were sequenced using PacBio HiFi long reads by CNRGV, INRA Occitanie Toulouse, France (cnrgv.toulouse.inra.fr). Circular consensus sequences were *de-novo* assembled in contigs using the hifiasm assembler with default parameters^77^. Subsequently, the contigs were ordered in ALLMAPS^78^ using a genetic map derived from the cross of inbred lines EP1xPH207 as reference^79^.

### Comparative genomic analyses

Genomic positions of KASP markers in the QTL region on chromosome 10 of KE0095 and KE0109 were obtained by mapping their probe sequences against KE0095 and KE0109 PacBio HiFi genomes using bwa-mem^80^. Genomic positions of B73v5 genes^75^ in KE0095 and KE0109 were identified using BLAST + 2.9.0^81^.

Pairwise sequence alignment of chromosome 10 between KE0095 and KE0109 was conducted using nucmer with a minimum length of a cluster set to 156 and a maximum gap between adjacent matches in a cluster set to 1000, and subsequently filtered using delta-filter with settings of minimum identity of 95% and minimum alignment length of 1000^82^.

To compare the amino acid sequences of candidate genes in the QTL region on chromosome 10 between KE0095 and KE0109, coding sequences of the candidate genes were extracted from the respective genome assembly. Coding sequences were translated to amino acids and aligned using Clustal Omega^83^.

LHCII components of *Arabidopsis thaliana* were identified in Araport 11 (Supplementary Table S7)^54^ and searched against B73v5 proteins^75^ via BLAST + 2.9.0^81^. The search result was filtered for minimum sequence identity of 70% and minimum query coverage of 75%. For each query, the search result with the highest bit score was selected and reported (Supplementary Table S7).

### Haplotype distribution in elite lines

Data for 136 diverse Dent and Flint inbred lines genotyped with the 600k Array were obtained from Unterseer et al.^31^ and merged with the 600k SNP data from the Kemater DH lines, retaining only SNPs present in both datasets. The 15 SNPs closest to *lhcb6* in B73v5 (8 upstream; 7 downstream), including the most significant SNP from the GWAS analysis (AX-90599221; lead SNP), were concatenated to define haplotype alleles. These haplotype alleles were then filtered to keep only those occurring at least three times in the combined dataset.

### Statistical analyses

All statistical analyses were performed in R (R Core Team, 2013). The statistical model for estimating genotype and genotype by environment variance components of F_2:3_ recombinant lines derived from the cross of the DH lines KE0095 and KE0109 in field experiments 2024 was:

$${y}_{tkos}=\mu+{\eta}_{t}+{g}_{k\left(t\right)}+{l}_{o}+g{l}_{ko\left(t\right)}+{r}_{s\left(o\right)}+{e}_{tkos}$$

where ${y}_{tkos}$ are the phenotypic observations; $\mu$ is the overall mean; ${\eta}_{t}$ is the fixed effect of the group $t$ ($t$ =1 for checks, $t$ =2 for F_2:3_ recombinant lines); ${g}_{k\left(t\right)}$ is the random effect of genotype $k$ nested within group$t$; ${l}_{o}$ is the random effect of environment $o$; $g{l}_{ko\left(t\right)}$ is the random interaction effect for genotype $k$ and environment $o$ nested within group $t$; ${r}_{s\left(o\right)}$ is the random effect of the block $s$ nested within environment $o$ and ${e}_{tkos}$ is the residual error. Genotypic and genotype by environment variance components were modeled individually for recombinant lines and for checks. Restricted maximum-likelihood estimation implemented in ‘ASRemlR’ (v4.1.0) was used for estimating variance components and their standard errors. Trait heritability (h^2^) and associated confidence intervals were calculated on an entry mean basis applying established procedures^84,85^.

Adjusted entry means for the F_2:3_ recombinant lines, the subset of 27 Kemater lines, and respective checks were calculated using the same model but dropping the group effect (${\eta}_{t}$) and treating genotype as a fixed effect. Adjusted means for KE0095, KE0109 and four NILs in the growth chamber experiments were calculated using a statistical model with genotype as fixed effect and block as random effect.

To evaluate the association between marker alleles and phenotypic data in the F_2:3_ recombinant lines, significance of marker effects was assessed for 14 markers (Supplementary Table S1) based on adjusted means with incremental Wald tests implemented in ‘ASRemlR’ (v4.1.0)^86^. Marker alleles were coded as 0 and 2 and marker effects were considered significant at a FDR of 1%. Phenotypic variance explained (PVE) by the markers was calculated as the coefficient of determination (R^2^) of the regression of F_v_/F_m_ adjusted means on marker alleles. Genetic variance explained (GVE) was calculated as PVE divided by the heritability estimate of F_v_/F_m_^87^. To assess the effect of the *lhcb6-B* allele on F_v_/F_m_ and early plant height in the subset of 27 Kemater lines, a two-way analysis of variance (ANOVA) was conducted with the genes *lhcb6* and *ndhm1*^18^, and their interaction as factors. Significance for the traits F_v_, F_m_, F_v_/F_m_, NPQ, fresh and dry biomass and half rise time from F_0_ to F_j_ in comparisons of KE0095, KE0109 and four NILs in the growth chamber experiments was tested with Student’s t-tests with Benjamini-Hochberg correction to control for multiple testing.

To assess the significance of differential protein accumulation in the 27 Kemater lines, an ANOVA was conducted for each protein separately. To avoid false positive associations, proteins were considered differentially accumulated at a FDR of 20%.

## Supplementary Information

Below is the link to the electronic supplementary material.

Supplementary Material 1

Supplementary Material 2

Supplementary Material 3

Supplementary Material 4

## Data Availability

The mass spectrometry proteomics data have been deposited to the ProteomeXchange Consortium via the PRIDE^88^ partner repository with the dataset identifiers PXD069740 and PXD070951.
