# Supplementary material for: Native alleles at lhcb6 shape photosynthetic efficiency and early growth in maize
Source: Sci Rep. 2026 Mar 6;16:8486. doi: 10.1038/s41598-026-42348-8 (PMC12972323; doi:10.1038/s41598-026-42348-8)
Supplement: Supplementary file 1 — Supplementary Material 1 [file 41598_2026_42348_MOESM1_ESM.pdf]

Native alleles at *lhcb6* shape photosynthetic efficiency and early growth in maize

Sebastian Urzinger 1†, Lukas Würstl 1†, Viktoriya Avramova 1, Claude Urbany 2, Daniela Scheuermann 2, Thomas Presterl 2, Stefan Reuscher 2, Manfred Mayer 1, 3, Sarah Brajkovic 4, Bernhard Küster 4, Milena Ouzunova 2, Bernardo Ordas 5, Peter Westhoff 6, Chris-Carolin Schön* 1

Supplement

Supplementary figures

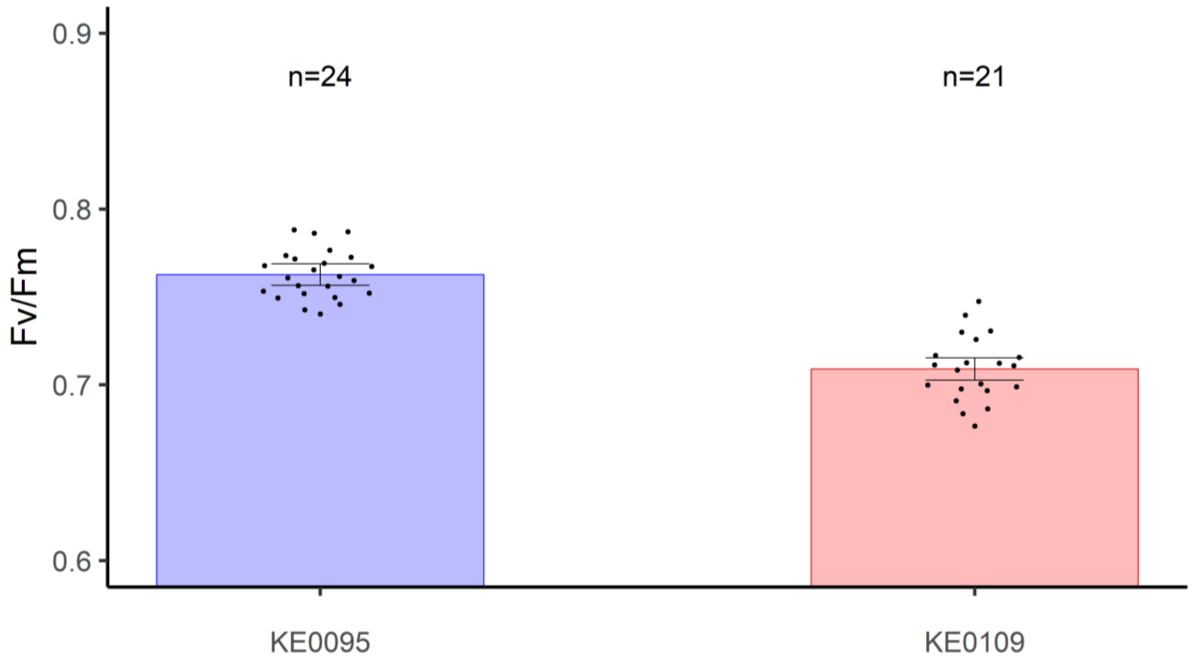

Supplementary Figure S1 Significant difference in F_v/F_m between the parental lines KE0095 and KE0109 of the biparental population evaluated in a growth chamber experiment. Bars represent adjusted means ± standard errors and dots represent individual plant observations.

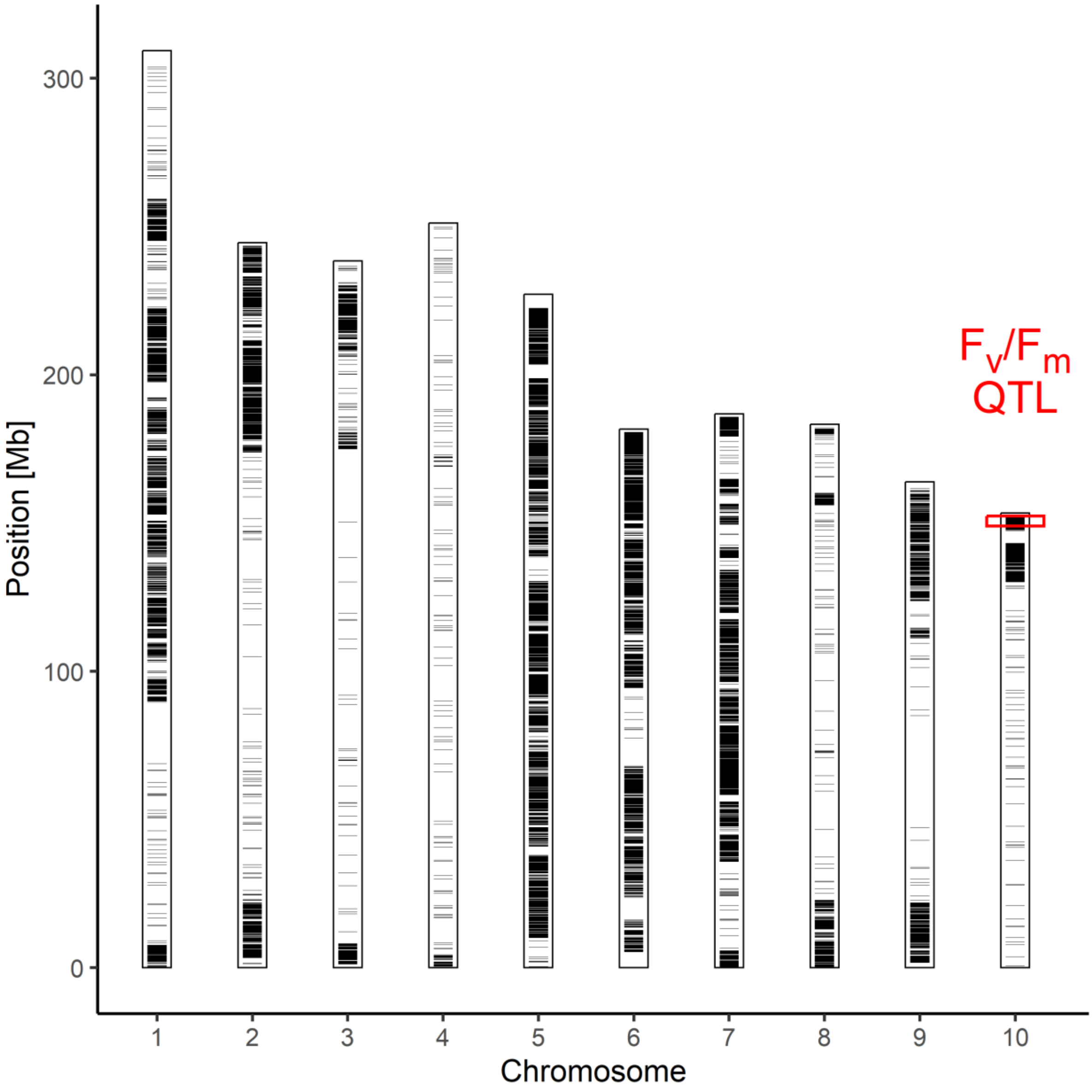

Supplementary Figure S2 Map position of 74,795 SNPs polymorphic between DH lines KE0095 and KE0109, the parental lines of the biparental population used for fine-mapping. The QTL for F_v/F_m on chromosome 10 is highlighted with a red box.

```
                            TATA box
B73          ----CG----GCTATTAT-TATTAATCTGACGTCCTGCTGCTGTTCCCTCCAATCCAACG      51
KE0095       ----GG----CTATTAAT-TATTAATCTGACGTCCTGCTGCTGTTCCCTCCAATCCAACG      51
KE0109       ATTGGGGTCAACCATGGTGAAAAAACATGGGGTATAAGTGATTTTGCCAA---------A      51
                  *        *  *  *  **  **  **     ** * ** **
                                                           start codon
B73          AACTGACCACAACCACCTAAGCTAAAAGCTAACCACCAGACCCCCGGCCATGGCGCTCGC      111
KE0095       AACTGACCACAACCACCTAAGCTAAAAGCTAACCACCAGACCCCCGGCCATGGCGCTCGC      111
KE0109       ATCGTTGGTGTCAGCCGACACCGATGACTCCACATAGAATCGCCCCTGCATGGCGCTCGC      111
             * *            *   * * *  *    **    *  * ***   ************

B73          GCCCACTTCCGCTGCCGTGCTCAAGACCCCGTTCCTCGGGGCCAGGCGCGCGCTCGCCAA      171
KE0095       GCCCACCTCCGCTGCCGTGCTCAAGACCCCGTTCCTCGGGGCCAGGCGCGCGCTTGCCAA      171
KE0109       GCCCACCTCCGCTGCCGTGCTCAAGACCCCGTTCCTCGGGGCCAGGCGCGCGCTTGCCAA      171
             ****** *********************************************** *****

B73          TGCCGCCGCCAAGCCCGCGCCTCGCCGCGCGCTGGTCGTCGCCGCCGCGGCCGCCGCCAA      231
KE0095       TGCCGCCGCCAAGCCCGCGCCTCGCCGCGCGCTCGTCGTCGCCGCCGCGGCCGCCGCCAA      231
KE0109       TGCCGCCGCCAAGCCCGCGCCTCGCCGCGCGCTGGTCGTCGCCGCCGCGGCCGCCGCCAA      231
             ********************************* **************************

B73          GAAGTCGTGGATCCCGGCCATCAAGAGCGACGCCGAGATCGTGAACCCGCCCTGGCTCGA      291
KE0095       GAAGTCGTGGATCCCGGCCATCAAGAGCGACGCCGAGATCGTGAACCCGCCCTGGCTCGA      291
KE0109       GAAGTCGTGGATCCCGGCCATCAAGAGCGACGCCGAGATCGTCAACCCGCCCTGGCTCGA      291
             ***************************************** ******************

B73          CGGCTCGTGAGTCTTTCGCTGTCTGTCTATGTTCACTTATAACTGTGATACGTACGAGGA      351
KE0095       CGGCTCGTGAGTCTTTCGCTGTCTGTCTATGTTCACTTATAACTGTGATACGTACGAGGA      351
KE0109       CGGCTCGTGAGTCTTTCGCTGTTTGTCTATGTTCACTTATAACTGTGATACGTACGAGGA      351
             ********************** *************************************

B73          GAAAAATTAAATGGAGGTCGAAGCTGACCACG--------------ACGACTTGCAGGCT      397
KE0095       GAAAAATTAAATGGAGGTCGAAGCTGACCACGACGAAGCGGCCGCGACGACTTGCAGGCT      411
KE0109       GAAAAATTAAATGGAGGTCGAAGCTGACCACG--------------ACGACTTGCAGGCT      397
             ********************************              **************

B73          CCCCGGCGACTTCGGGTTCGACCCGCTGGGGCTGGGCAAGGACCCGGCGTTCCTCAAGTG      457
KE0095       CCCCGGCGACTTCGGGTTCGACCCGCTGGGGCTGGGCAAGGACCCGGCGTTCCTCAAGTG      471
KE0109       CCCCGGCGACTTCGGGTTCGACCCGCTGGGGCTGGGCAAGGACCCGGCGTTCCTCAAGTG      457
             ************************************************************

B73          GTACCGGGAGGCGGAGCTGATCCACGGGCGGTGGGCGATGGCGGCCGTGCTGGGCATCTT      517
KE0095       GTACCGGGAGGCGGAGCTGATCCACGGGCGGTGGGCGATGGCGGCCGTGCTGGGCATCTT      531
KE0109       GTACCGGGAGGCGGAGCTGATCCACGGGCGGTGGGCGATGGCGGCCGTGCTGGGCATCTT      517
             ************************************************************

B73          CGTGGGGCAGGCGTGGAGCGGCATCCCGTGGTTCGAGGCCGGCGCGGACCCGAGCGCCAT      577
KE0095       CGTGGGGCAGGCGTGGAGCGGCATCCCGTGGTTCGAGGCCGGCGCGGACCCGAGCGCCAT      591
KE0109       CGTGGGGCAGGCGTGGAGCGGCATCCCGTGGTTCGAGGCCGGCGCGGACCCGAGCGCCAT      577
```

Supplementary Figure S3 Comparison of *lhcb6* genomic sequences from B73, KE0095 and KE0109. The B73 genomic DNA sequence was obtained from MaizeGDB (B73v5) for the gene model *Zm00001eb433540* [1]. The corresponding genomic sequences for KE0095 and KE0109 were identified via BLAST using the coding sequence of *Zm00001eb433540*, including 100 bp upstream of the start codon. In the alignment, the putative TATA box is highlighted in red, and the start codon is marked in blue.

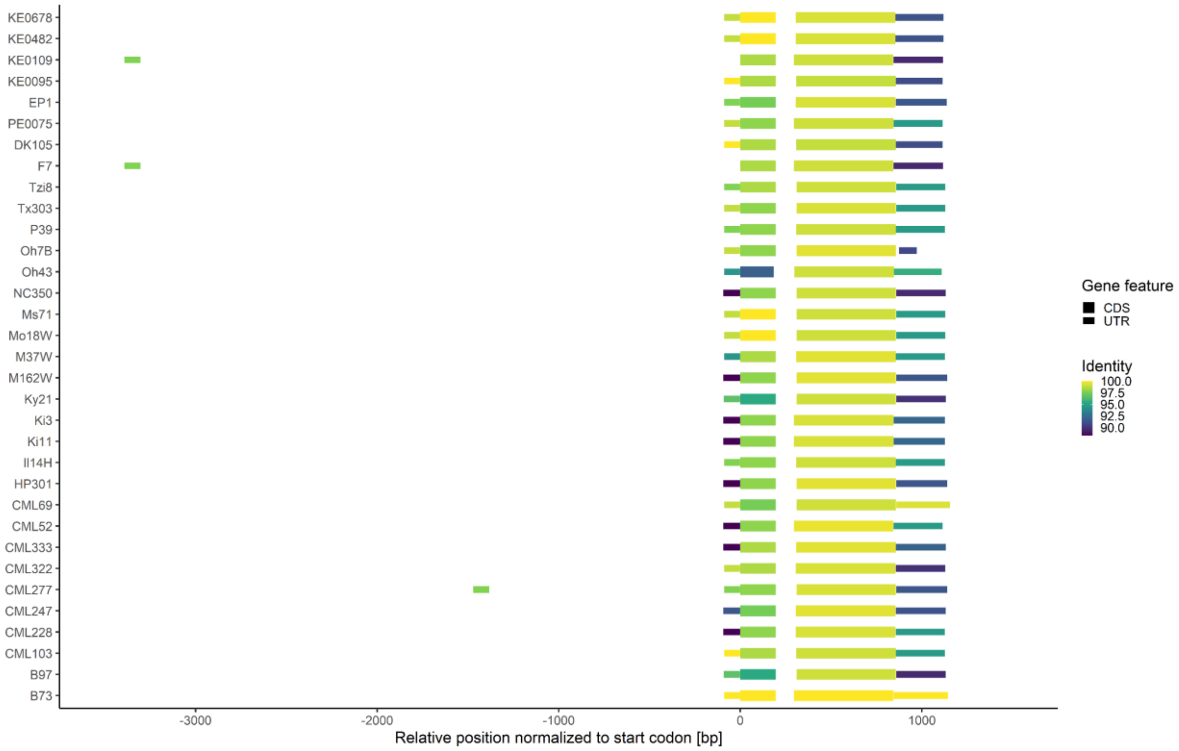

Supplementary Figure S4 Structural comparison of *lhcb6* gene models across diverse maize genome assemblies. Genomic sequences corresponding to the 3′ UTR, 5′ UTR, and coding sequences (CDS) of *lhcb6* from B73v5 (Zm00001eb433540) [1] were used as queries in BLAST searches against each genome assembly. For each gene feature, only the top BLAST hit was retained, and its position was normalized relative to the start codon. Colors indicate the level of identity with the respective B73 gene feature.

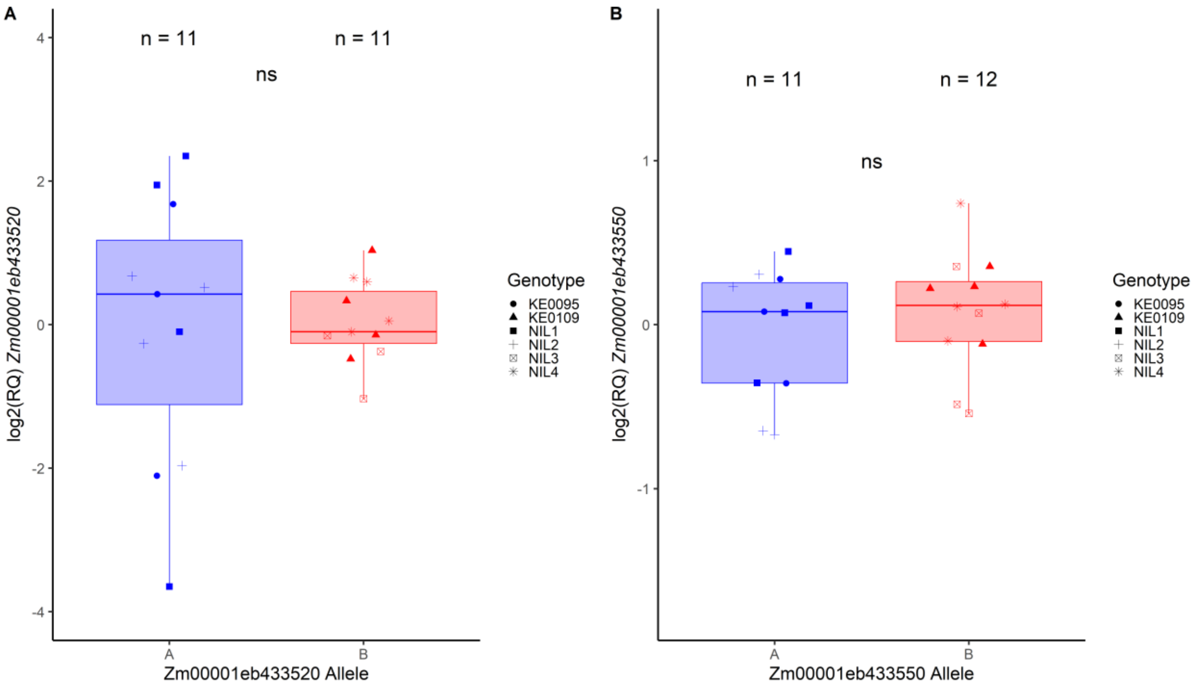

Supplementary Figure S5 Transcript levels of candidate genes *Zm00001eb433520* and *Zm00001eb433550*. A, B) Log2-transformed relative transcript levels of *Zm00001eb433520* **(A)** and *Zm00001eb433550* **(B)** in near isogenic lines (NILs) with contrasting *lhcb6* alleles and of parental lines KE0095 and KE0109. Boxplots with median as center line, box limits indicate upper and lower quartiles and whiskers 1.5× interquartile range. Statistical differences were assessed by analyses of variance (ANOVA). ns: not significant.

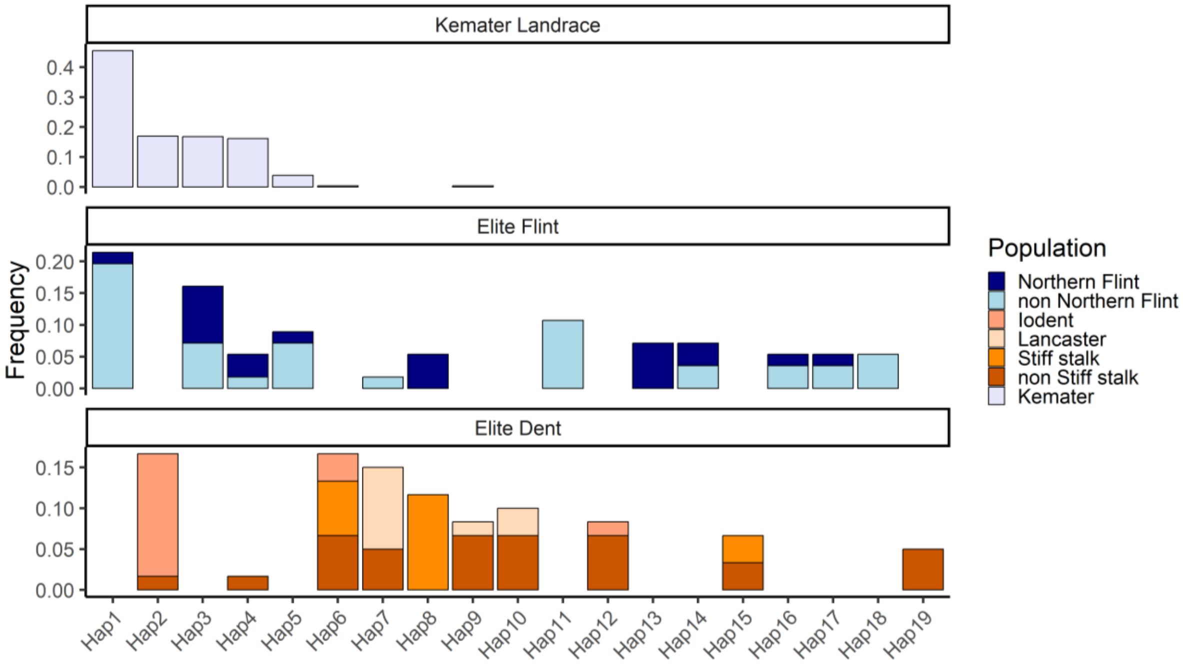

Supplementary Figure S6 Diversity of *lhcb6* haplotypes in inbred maize lines and the landrace Kemater. Haplotypes were defined by concatenating the 15 SNPs closest to *lhcb6* from the 600k Axiom™ Maize Genotyping Array [2] and filtered to include only those occurring at least three times. Genotyping data and population assignments for 136 inbred lines were obtained from Unterseer et al. [3]. The Kemater landrace and heterotic groups of elite lines are indicated by colors.

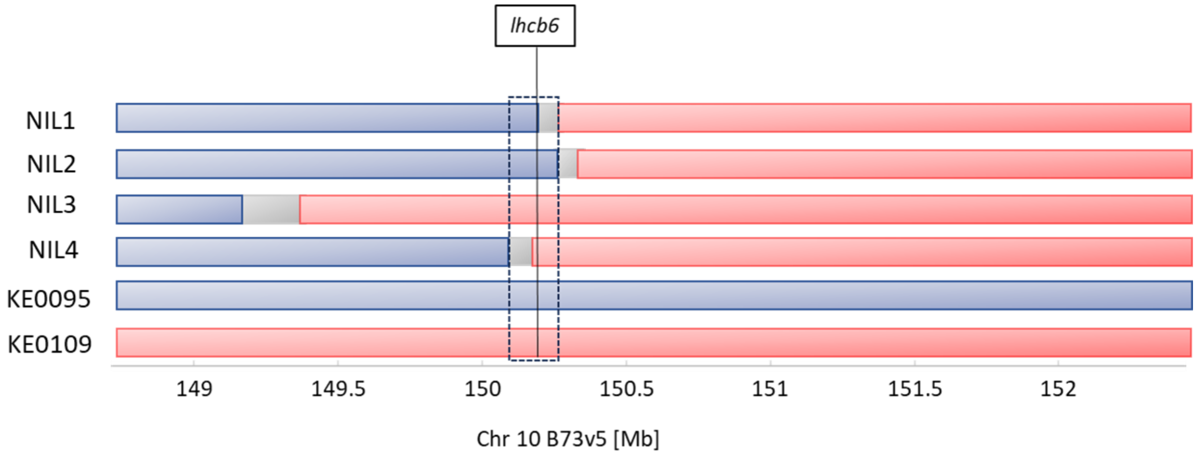

Supplementary Figure S7 Genetic composition of near isogenic lines (NILs) and doubled-haploid (DH) parental lines used for physiological analysis in controlled conditions. Genomic regions carrying the allele of the recurrent parent (KE0095) are shown in blue, while those with the donor parent (KE0109) allele are shown in red. A 154 kb region distinguishing NIL 1 and NIL 2 from NIL 3 from NIL 4 and containing 13 gene models in the B73v5 reference genome is highlighted with a dashed box. The position of the gene *lhcb6* (*light-harvesting chlorophyll a/b binding protein6*, *Zm00001eb433540*) is marked with a vertical line.

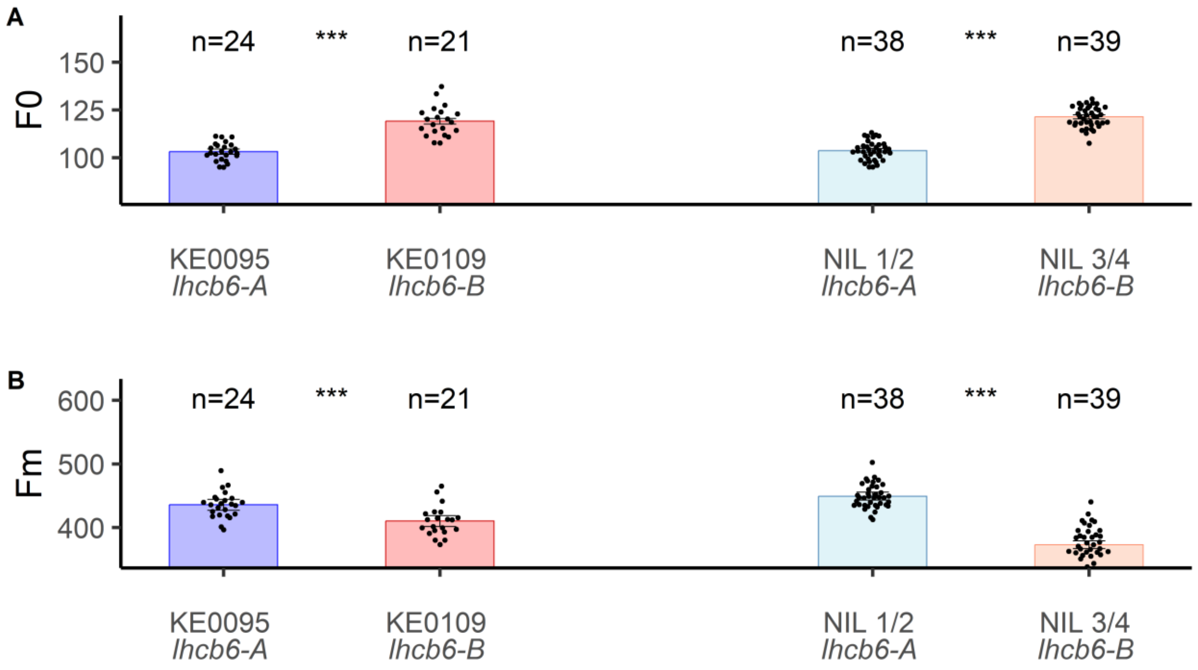

Supplementary Figure S8 Minimal (F_0) and maximal (F_m) fluorescence of near isogenic lines (NILs) with contrasting *lhcb6* alleles and their parents in a growth chamber experiment. A) F_0 of dark-adapted leaves. **B**) F_m after applying a saturating light flash to the dark-adapted leaves. Measurements were taken at growth stages V5–V6. Significant differences based on Student's t-tests are indicated by stars. ** $p < 0.01$; ***$p < 0.001$. Bars represent adjusted means ± standard error and dots represent individual plant observations.

Supplementary tables

Supplementary Table S1 Genomic positions of KASP markers used to genotype $F_{2:3}$ recombinant lines and near isogenic lines (NILs) from the cross of KE0095 and KE0109. Positions were determined by mapping the probes of the publicly available Axiom™ Maize 600k Genotyping Array **[2]** to the respective genome assembly using the bwa-mem alignment tool[4].

**Marker**	**Chromosome**	**Position B73v5**	**Position KE0095**	**Position KE0109**	**Genotyped in:**
AX-90560736	10	143,112,717	143,319,871	142,274,063	NIL
AX-90598666	10	149,378,445	148,740,234	148,284,863	$F_{2:3}$, NIL
AX-91370210	10	149,463,669	148,872,430	148,442,955	$F_{2:3}$, NIL
AX-90611044	10	149,568,533	148,960,964	148,502,800	$F_{2:3}$, NIL
AX-90560838	10	149,660,591	149,098,879	148,675,336	$F_{2:3}$, NIL
AX-91365760	10	149,850,831	149,301,235	148,822,092	$F_{2:3}$, NIL
AX-91196016	10	149,960,879	149,413,540	148,934,912	$F_{2:3}$, NIL
AX-91369070	10	150,109,398	149,571,593	149,028,281	$F_{2:3}$, NIL
AX-90599221	10	150,176,073	149,731,916	149,097,971	$F_{2:3}$, NIL
AX-91196092	10	150,262,989	149,805,780	149,172,008	$F_{2:3}$, NIL
AX-90564314	10	150,367,233	149,893,811	149,194,873	$F_{2:3}$, NIL
AX-91196161	10	150,507,597	150,023,962	149,341,009	$F_{2:3}$, NIL
AX-90560860	10	150,712,585	150,262,052	149,463,226	$F_{2:3}$, NIL
AX-91196242	10	150,804,175	150,371,931	149,580,278	$F_{2:3}$, NIL
AX-90531911	10	150,914,661	151,359,910	150,249,567	$F_{2:3}$, NIL
AX-91196351	10	151,042,161	150,667,619	149,852,738	NIL
AX-90577486	10	151,152,169	151,359,910	150,249,567	NIL
AX-91834732	10	151,308,012	151,558,978	150,439,327	NIL
AX-91365786	10	151,404,551	151,616,333	150,487,914	NIL
AX-91196558	10	151,738,373	151,883,122	150,922,091	NIL
AX-91834854	10	151,941,918	152,085,768	151,062,065	NIL
AX-91834882	10	152,116,439	152,337,249	151,249,867	NIL
AX-91365808	10	152,307,284	152,503,216	151,453,405	NIL
AX-91196698	10	152,357,073	152,552,940	151,512,733	NIL

Supplementary Table S2 Candidate genes in the 154 kb fine-mapped region for F_v/F_m on chromosome 10. The B73v5 identifiers [1] of candidate genes in the fine-mapped region for F_v/F_m, along with their annotations from NCBI, are listed. Two of the candidate genes were identified in the leaf proteomes of the parents of the bi-parental mapping population (KE0095, KE0109) and in a set of 27 Kemater lines differing at their *lhcb6* allele. Differences in protein accumulation were tested using two-sided Student's t-tests. ns: no significant differences in protein levels between groups. ****p* < 0.001; nd: not detected.

**Gene**	**Annotation**	**Proteomics KE0095 - KE0109**	**Proteomics 27 Kemater**
Zm00001eb433490	*Putative homeodomain-like transcription factor superfamily protein*	nd	nd
Zm00001eb433500	*crr2 - Cytokinin response regulator2*	nd	nd
Zm00001eb433510	*Early nodulin-like protein1*	nd	nd
Zm00001eb433520	*Uncharacterized protein*	nd	nd
Zm00001eb433530	*ubiquinone biosynthesis protein COQ4 homolog mitochondrial*	nd	nd
Zm00001eb433540	*lhcb6 - light harvesting chlorophyll a/b binding protein6*	***	***
Zm00001eb433550	*pat38 - protein S-acyltransferase38*	nd	nd
Zm00001eb433560	*Uncharacterized protein*	nd	nd
Zm00001eb433570	*Thioredoxin domain-containing protein*	nd	nd
Zm00001eb433590	*Uncharacterized protein*	nd	nd
Zm00001eb433600	*FAD-binding PCMH-type domain-containing protein*	nd	nd
Zm00001eb433610	*chlh1 - Mg chelatase subunit H 1*	ns	ns
Zm00001eb433620	*ysl10 - yellow stripe-like transporter10*	nd	nd

Supplementary Table S3 Polymorphisms between KE0095 and KE0109 in genes within the 154 kb fine-mapped region for F_v/F_m on chromosome 10. For each SNP, the nucleotide of KE0095, KE0109 and B73v5 [1] at the respective position is provided, along with the predicted amino acid exchange and whether the exchange is considered conservative.

Gene	KE0095	KE0109	B73v5	Predicted change	Nature of amino acid exchange	Annotation
Zm00001eb433500	T	G	G	S→A	conservative	*crr2 - Cytokinin response regulator2*
	T	C	C	synonymous		
	G	C	C	synonymous		
	C	T	C	synonymous		
	G	C	C	synonymous		
	G	C	G	synonymous		
	G	A	G	synonymous		
	G	C	G	synonymous		
Zm00001eb433510	T	G	G	synonymous		*Early nodulin-like protein1*
	A	G	A	T→A	conservative	
	A	G	G	synonymous		
Zm00001eb433520	A	-	A	Q→-		Uncharacterized protein
	G	-	G			
	C	-	C			
	-	T	-	-→A		
	-	G	-			
	-	G	-			
	-	C	-	-→A		
	-	G	-			
	-	G	-			
	-	C	-	-→L		
	-	G	-			
	-	C	-			
	T	C	T	synonymous		
	G	C	G	V→L	conservative	
	G	-	G	G→-		
	C	-	C			
	G	-	G			
	C	-	C	A→-		
	C	-	C			
	G	-	G			
	A	-	A	D→-		
	C	-	C			
	C	-	C			
	C	-	C	P→-		
	T	-	T			
	G	-	G			
	C	-	C	A→-		
	C	-	C			
	G	-	G			
	T	-	T	V→-		
	C	-	C			
	G	-	G			
	A	C	A	synonymous		
	-	T	-	-→S		
	-	C	-			
	-	G	-			
	C	G	C	G→A	conservative	
	G	C	G	synonymous		
	A	G	A	synonymous		
	A	G	A	synonymous		

	C	T	C	A→V	non-conservative	
	T	A	T	S→T	conservative	
	C	A	C	L→I	conservative	
	C	G	C	L→V	conservative	
	T	C	T	V→A	non-conservative	
	A	G	A	synonymous		
	C	G	C	synonymous		
	A	C	A	D→A	conservative	
	A	C	A	synonymous		
	C	T	C	A→V	non-conservative	
	T	C	T	V→A	non-conservative	
	T	G	T	synonymous		
Zm00001eb433530	T	A	T	synonymous		*ubiquinone biosynthesis protein COQ4 homolog mitochondrial*
	C	G	C	Q→E	conservative	
	T	C	T	synonymous		
	G	T	G	synonymous		
Zm00001eb433550	A	G	A	E→G	conservative	*pat38 - protein S-acyltransferase38*
	G	A	A	S→N	conservative	
	A	G	G	synonymous		
	A	G	A	synonymous		
	C	T	T	synonymous		
	A	G	A	synonymous		
	G	A	G	S→N	conservative	
	C	T	T	T→I	non-conservative	
	C	T	C	synonymous		
	G	C	C	synonymous		
	G	A	A	synonymous		
	T	C	C	synonymous		
	G	A	A	synonymous		
	-	C	C	-→S		
	-	A	A			
	-	T	T			
	G	C	C	G→R	conservative	
	T	C	C	M→T	conservative	
	T	G	G	I→M	conservative	
	C	T	T	synonymous		
	C	A	C	L→M	conservative	
Zm00001eb433620	G	T	T	synonymous		*ysl10 - yellow stripe-like transporter10*
	C	T	T	synonymous		
	T	C	C	synonymous		
	T	C	C	synonymous		
	T	C	C	synonymous		
	T	C	C	synonymous		
	T	C	C	synonymous		

Supplementary Table S4 Validation set of 27 Kemater lines used for proteomic analysis and their allele at AX-90599221 (lead SNP from GWAS) and *lhcb6*. The allelic status of *lhcb6* was determined by amplifying a genomic fragment containing a hAT transposon insertion in the putative promoter of *lhcb6-B*. The *lhcb6* haplotype was determined by concatenating the 15 SNPs closest to *lhcb6* from the Axiom™ Maize 600k Genotyping Array [2].

Genotype	AX-90599221	PCR Genotyping	*Lhcb6* haplotype
KE0007	Ade	*lhcb6-A*	Hap2
KE0011	Gua	*lhcb6-B*	Hap1
KE0045	Ade	*lhcb6-A*	Hap3
KE0054	Ade	*lhcb6-A*	Hap2
KE0067	Gua	*lhcb6-B*	Hap1
KE0079	Gua	*lhcb6-B*	Hap1
KE0096	Ade	*lhcb6-A*	Hap4
KE0103	Gua	*lhcb6-B*	Hap1
KE0134	Gua	*lhcb6-B*	Hap1
KE0153	Ade	*lhcb6-A*	Hap4
KE0190	Gua	*lhcb6-B*	Hap1
KE0194	Gua	*lhcb6-B*	Hap1
KE0202	Ade	*lhcb6-A*	Hap3
KE0204	Ade	*lhcb6-A*	Hap4
KE0241	Gua	*lhcb6-B*	Hap1
KE0277	Gua	*lhcb6-B*	Hap1
KE0288	Ade	*lhcb6-A*	Hap3
KE0294	Gua	*lhcb6-B*	Hap1
KE0407	Gua	*lhcb6-B*	Hap1
KE0426	Ade	*lhcb6-A*	Hap5
KE0462	Ade	*lhcb6-A*	Hap3
KE0482	Ade	*lhcb6-A*	Hap5
KE0557	Ade	*lhcb6-A*	Hap4
KE0590	Ade	*lhcb6-A*	Hap3
KE0627	Gua	*lhcb6-B*	Hap1
KE0633	Ade	*lhcb6-A*	Hap3
KE0678	Ade	*lhcb6-A*	Hap5

Supplementary Table S5. Summary of linear model results assessing the effects of *lhcb6* and *ndhm1* on early growth traits in 27 Kemater lines. The factors *lhcb6* and *ndhm1* represent the allelic states of each gene. *lhcb6* has two levels (*lhcb6-A*, n = 15; *lhcb6-B*, n = 12), as well as *ndhm1* (*ndhm1-A1*, n = 15; *ndhm1-A2*, n = 12). Genotypic data for *ndhm1* were derived from Urzinger et al. [5]. Significance of the terms was assessed by a two-way analysis of variance (ANOVA). The table reports effect estimates, standard errors (SE), degrees of freedom (Df), sum of squares (SS), F-values, *p*-values, and model R^2.

Phenotype	Term	Estimate	SE	Df	SS	F	*p*-value	R^2
**F_v/F_m (V6)**	Intercept	0.770	0.003					0.74
	Lhcb6-B	-0.018	0.006	1	2.33×10^{-3}	19.94	1.76×10^{-4}	
	Ndhm1-A2	-0.034	0.006	1	5.03×10^{-3}	43.06	1.07×10^{-6}	
	Lhcb6-B: Ndhm1-A2	0.013	0.009	1	2.53×10^{-4}	2.17	0.15	
	Residuals			23	2.68×10^{-3}			
**PH (V6)**	Intercept	62.47	1.58					0.48
	Lhcb6-B	-1.65	2.73	1	153.69	6.19	2.05×10^{-2}	
	Ndhm1-A2	-6.48	2.73	1	371.40	14.96	7.80×10^{-4}	
	Lhcb6-B: Ndhm1-A2	-2.62	3.99	1	107.08	0.43	0.52	
	Residuals			23	570.82			

Supplementary Table S6 Introgressions from KE0109 in near isogenic lines (NILs) derived from the cross KE0095 x KE0109. Positions of KASP markers were determined by mapping the probes of the publicly available Axiom™ Maize 600k Genotyping Array [2] to the respective genome assembly using the bwa-mem[4] alignment tool. AX-91196698 is the most distal KASP marker on chromosome 10.

**NIL**	**Start of introgression from KE0109 between KASP markers**	**Positions [Chr 10 B73v5]**	**End of introgression from KE0109 between KASP marker and the end of chromosome 10**	**Positions [Chr 10 B73v5]**	***lhcb6* allele**
**NIL 1**	AX-90599221 & AX-91196092	150,176,073 & 150,262,989	AX-91196698	152,357,073 & 152,435,371	*lhcb6*-A
**NIL 2**	AX-91196092 & AX-90564314	150,262,989 & 150,367,233	AX-91196698	152,357,073 & 152,435,371	*lhcb6*-A
**NIL 3**	AX-90525780 & AX-91385859	149,259,081 & 149,374,786	AX-91196698	152,357,073 & 152,435,371	*lhcb6*-B
**NIL 4**	AX-91369070 & AX-90599221	150,109,398 & 150,176,073	AX-91196698	152,357,073 & 152,435,371	*lhcb6*-B

Supplementary Table S7 Identification of LHCII components based on homology to *Arabidopsis thaliana*. LHCII components described in *A. thaliana* were searched against proteins annotated in B73v5 [1] using protein BLAST [6]. Statistical significance of differences between Kemater lines with *lhcb6-A* (N = 15) and *lhcb6-B* (N = 12) was assessed using two-sided Student's t-tests, with resulting *p*-values adjusted by Bonferroni correction. Proteins that could not be distinguished by unique peptides are grouped into a single protein group. FC: fold change between *lhcb6-A* and *lhcb6-B* groups. nd: not detected

Identified Protein group (B73v5)	LHC component	log2(FC) 27 Kemater	*p*-value adjusted 27 Kemater	Best Arabidopsis hit
Zm00001eb357740; Zm00001eb161390	LHCB1	-0.4	1	AT2G34420.1 (LHCB1.5)
Zm00001eb343900	LHCB1	-0.2	1	AT2G34420.1 (LHCB1.5)
Zm00001eb161410	LHCB1	nd	nd	AT2G34420.1 (LHCB1.5)
Zm00001eb049630; Zm00001eb049640	LHCB2	0.1	1	AT2G05100.1 (LHCB2.1)
Zm00001eb324240	LHCB3	-0.9	8.6E-05	AT5G54270.1 (LHCB3)
Zm00001eb106810	LHCB4	-1.5	0.84	AT5G01530.1 (LHCB4.1)
Zm00001eb324090	LHCB4	-0.3	1	AT5G01530.1 (LHCB4.1)
Zm00001eb112260	LHCB5	0.2	1	AT4G10340.1 (LHCB5)
Zm00001eb168100	LHCB5	0.0	1	AT4G10340.1 (LHCB5)
Zm00001eb066480	LHCB6	0.2	1	AT1G15820.1 (LHCB6)
Zm00001eb433540	LHCB6	-4.0	1.2E-12	AT1G15820.1 (LHCB6)

Supplementary Table S8 Differentially accumulated proteins in 27 Kemater lines grouped by their *lhcb6* allele. The B73v5 identifiers [1] of differentially accumulated proteins and their associated gene ontology (GO) terms are provided. Proteins are listed in order of significance of differential accumulation. The significance of differential accumulation was determined by analyses of variance (ANOVA), with proteins selected based on a false discovery rate < 20% [7]. FC: fold change between *lhcb6-A* and *lhcb6-B* groups.

B73v5	Annotation	log2(FC)	Biological Process	Cellular Component	Molecular Function
Zm00001eb433540	LHCB6	-4.0	photosynthesis, light harvesting; protein-chromophore linkage; response to light stimulus	photosystem I; photosystem II; chloroplast thylakoid membrane; plastoglobule; chloroplast envelope; integral component of membrane	chlorophyll binding; protein domain specific binding; metal ion binding; mRNA binding
Zm00001eb289810		-1.5		Golgi apparatus; integral component of membrane	
Zm00001eb324240	LHCB3	-0.9	photosynthesis, light harvesting; protein-chromophore linkage; response to light stimulus; regulation of stomatal movement; response to herbicide; response to abscisic acid	photosystem I; photosystem II; chloroplast thylakoid membrane; plastoglobule; light-harvesting complex; chloroplast envelope; integral component of membrane	chlorophyll binding; protein domain specific binding; metal ion binding
Zm00001eb068760	KAS III	-0.5	fatty acid biosynthetic process	chloroplast; plastid stroma; integral component of membrane	3-oxoacyl-[acyl-carrier-protein] synthase activity; beta-ketoacyl-acyl-carrier-protein synthase III activity
Zm00001eb271080		1.4	intermembrane lipid transfer; ceramide 1-phosphate transport	cytoplasm; membrane; cell periphery	lipid transfer activity; ceramide 1-phosphate binding; sphingolipid transporter activity; phospholipid transporter activity
Zm00001eb039350	CAO1	1.8	chlorophyll catabolic process	integral component of membrane	chlorophyllase activity; pheophytinase b activity

Supplementary Table S9 Primer used for RT-qPCR analysis and genotyping different *lhcb6* alleles. Elongation time was 30 sec for the PCRs and 60 sec for RT-qPCRs.

**Primer Name**	**Sequence**	**Application**	**Annealing T**
lhcb6_3UTR_FW	CTC CTA GGG ACG TAC GGC T	*lhcb6* RT-qPCR	60°C
lhcb6_3UTR_RV	GCA CGC TAA ACC ACC AAC AC		
Mep_qRT_FW	TGT ACT CGG CAA TGC TCT TG	Housekeeping RT-qPCR	58°C
Mep_qRT_RV	TTT GAT GCT CCA GGC TTA CC		
Zm00001eb433520_FW	CTA GTT GCA TCA TCT TGG GTA CG	*Zm00001eb433520* RT-qPCR	62°C
Zm00001eb433520_RV	TTG TTA TGT GCC GCG AAA CTA C		
Zm00001eb433550_FW	CAG ACT GCA AGG AAA CCT GAT AGT A	*Zm00001eb433550* RT-qPCR	62°C
Zm00001eb433550_RV	CTG AGA AAA CAA CAG GAT ATA CCG C		
fwd_Lchb6	TTT TAC CTC CCA ATC AAC TTT CTC AAA A	Discrimination *lhcb6-A* / *lhcb6-B*	65°C
rev_Lchb6	CTC TTG ATG GCC GGG ATC		

References

1 Hufford, M. B., *et al.* De novo assembly, annotation, and comparative analysis of 26 diverse maize genomes. *Science* **373**, 655–662 (2021).

2 Unterseer, S., *et al.* A powerful tool for genome analysis in maize: development and evaluation of the high density 600 k SNP genotyping array. *BMC Genomics* **15**, 1–15 (2014).

3 Unterseer, S., *et al.* A comprehensive study of the genomic differentiation between temperate Dent and Flint maize. *Genome biology* **17**, 1–14 (2016).

4 Li, H. & Durbin, R. Fast and accurate short read alignment with Burrows–Wheeler transform. *Bioinformatics* **25**, 1754–1760 (2009).

5 Urzinger, S., *et al.* Embracing native diversity to enhance the maximum quantum efficiency of photosystem II in maize. *Plant Physiology* **197**, kiae670 (2025).

6 Camacho, C., *et al.* BLAST+: architecture and applications. *BMC Bioinformatics* **10**, 421 (2009). https://doi.org/10.1186/1471-2105-10-421

7 Benjamini, Y. & Hochberg, Y. Controlling the false discovery rate: a practical and powerful approach to multiple testing. *Journal of the Royal Statistical Society: Series B (Methodological)* **57**, 289–300 (1995).
